# Optimal dose and type of exercise to improve cognitive function in adults with major depressive disorder: a systematic review and Bayesian model-based network meta-analysis

**DOI:** 10.3389/fpubh.2025.1662778

**Published:** 2025-12-19

**Authors:** Jiarong Ge, Qiyi Wang, Shimeng Wang, Xuan Xiong, Yifei Zhai

**Affiliations:** 1 Sports Science Institution, Nanjing University, Nanjing, Jiangsu, China; 2 College of Physical Education, Yangzhou University, Yangzhou, Jiangsu, China; 3 Institute of Sports Science, Nantong University, Nantong, Jiangsu, China; 4 Department of Physical Education, Nanjing University, Nanjing, Jiangsu, China

**Keywords:** exercise, cognition, major depressive disorder, dose–response, Bayesian model

## Abstract

**Introduction:**

Cognitive impairment is a core feature of major depressive disorder (MDD) that often persists during remission, significantly affecting psychosocial functioning. While exercise is known to alleviate depressive symptoms, its specific impact on cognitive domains remains variable. This study aims to evaluate the dose–response relationship and comparative effectiveness of different types of exercise interventions on cognitive function in adults with MDD.

**Methods:**

Five electronic databases (Ovid Medline, PsycINFO, Web of Science, Cochrane Central, and Embase) were systematically searched on September 20, 2024.

**Study selection:**

Randomized controlled trials (RCTs) involving adults aged 18 years or older with MDD were included if they examined the effects of exercise interventions and reported at least one cognitive outcome. Two independent reviewers conducted data extraction and quality assessment according to the Preferred Reporting Items for Systematic Reviews and Meta-Analyses for Network Meta-Analyses (PRISMA-NMA) guidelines.

**Results:**

A three-level meta-analysis and a Bayesian model-based network meta-analysis were performed. Fifteen RCTs with a total of 1,196 participants were included. A non-linear dose–response relationship was observed, with a minimum effective dose of 967 METs-min/week—within the WHO-recommended exercise range. Among exercise types, Tai Chi/Qigong demonstrated the greatest cognitive benefits. Moderation analysis indicated that younger age and a higher proportion of female participants were associated with stronger cognitive effects.

**Conclusion:**

Exercise significantly improves cognitive function in adults with MDD, with aerobic and mind–body exercises offering particularly strong benefits. These results support the integration of tailored exercise programs into treatment strategies for cognitive dysfunction in MDD.

**Systematic review registration:**

The systematic review was registered in PROSPERO (CRD42024546289): https://www.crd.york.ac.uk/prospero/display_record.php?ID=CRD42024546289.

## 1 Introduction

Major depressive disorder (MDD) is among the most common and debilitating neuropsychiatric disorders (1). Low mood, diminished interest or enjoyment in previous activities (2), and frequent suicidal or death-related thoughts (3) are all signs of MDD. It significantly increases health burdens, disability, and suicidality worldwide, affecting approximately 5% of adults globally (4, 5). Notably, cognitive function (CF) plays a critical role in depression, individuals with MDD often report experiencing cognitive impairments (6). This condition often persists even after symptom remission (7), regardless of the circumstances, improving cognitive impairments will be a key focus in the treatment of depression (8, 9). Cognitive impairment is highly prevalent among individuals with MDD, affecting approximately 40–60% of patients during acute episodes and often persisting into remission phases. These deficits primarily involve attention, executive function, and memory, which are closely related to daily functioning and relapse risk. Increasing evidence suggests that such cognitive disturbances are not merely secondary symptoms but represent a core and enduring feature of MDD, contributing to poor treatment outcomes and psychosocial dysfunction (10).

Some studies suggest cognitive deficits recover after remission (10), while others indicate they persist, potentially contributing to recurrent depression (11). Depressed individuals with cognitive impairment experience reduced quality of life, interpersonal dysfunction, and limited academic productivity (12), and antidepressants offer limited cognitive benefits with significant side effects (13). This underscores the need for effective non-pharmacological interventions to address cognitive impairments in depression.

Exercise, as a low-cost and low-side-effect intervention, has been effective in alleviating depression for over 30 years (14). Recent studies have emphasized the cognitive advantages of physical activity, particularly aerobic exercise, which has been demonstrated to enhance memory and executive functioning in individuals with depression (15). However, the impact of exercise on cognition may depend on the type of exercise (16). For example, Tai Chi combined with escitalopram has demonstrated significant cognitive improvements, as has resistance training, which is particularly beneficial for slowing cognitive decline in older adults (17).

Several recent systematic reviews and meta-analyses have explored the cognitive effects of exercise in depression (18, 19). However, most of these studies primarily compared different exercise interventions without exploring dose–response patterns or accounting for dependencies among multiple cognitive outcomes within trials. Moreover, few adopted a Bayesian analytical framework, which enables probabilistic estimation of optimal exercise doses and comparative rankings across modalities. Our study extends the existing literature by integrating three-level meta-analysis and Bayesian model-based dose–response network meta-analysis to comprehensively quantify how exercise dosage and type influence cognitive function in adults with MDD.

Nevertheless, the aforementioned studies overlooked two key priorities when using exercise interventions as treatment methods. First, the type of exercise may moderate the exercise-cognition relationship. Resistance exercise has also been found to enhance cognitive outcomes (20). Second, exercise prescriptions based on the FITT principle (i.e., frequency, intensity, time, and type) are frequently employed to evaluate how each exercise component affects cognitive development. However, considering these exercise characteristics in isolation may overlook the complex interactions between these factors. Additionally, the World Health Organization (WHO) 2020 Guidelines Development Group for Physical Activity and Sedentary Behavior also emphasizes that mood dysregulation in depression may hinder exercise adherence (21, 22), thus the need to determine the optimal and safe exercise dose for cognitive benefits in depressed individuals is necessary.

Despite growing recognition of depression as a leading contributor to the global disease burden, research investigating the cognitive dimension of depression remains fragmented and inconclusive (7). Existing meta-analyses have largely focused on symptom reduction rather than cognitive restoration, leaving a critical knowledge gap in understanding how exercise interventions may optimize cognitive outcomes in individuals with MDD. Furthermore, most prior studies have relied on simplified comparisons between exercise and control groups without addressing the nuanced dose–response patterns that could inform personalized exercise prescriptions (23). Given the World Health Organization’s call for scalable and sustainable strategies to improve mental and cognitive health worldwide, a comprehensive synthesis that quantifies the relationship between exercise parameters and cognitive improvement is urgently needed. This study therefore provides a timely contribution by integrating advanced meta-analytic approaches to clarify how different exercise modalities and doses can be optimized to support cognitive recovery in depression.

Overall, in order to address multiple effect sizes and retrieve pertinent randomized controlled trials, this study employs two innovative meta-analysis techniques: three-level meta-analysis and model-based dose–response network meta-analysis in a Bayesian framework. It aims to explore the dose–response relationship between exercise and CF in depressed patients and identify the optimal exercise dosage and type.

## 2 Methods

This study protocol was preregistered (PROS-PERO reference number #CRD42024546289) and all study procedures have followed guidelines recommended by the Preferred Reporting Items for Systematic Reviews and Network Meta-Analyses (PRISMA-NMA checklist) (24).

### 2.1 Search strategy and selection criteria

Five electronic databases were searched for relevant literature (e.i., Ovid Medline, PSYCINFO, Web of Science, Cochrane Central, and Embase) up to September 2024 (see Supplementary File 1). Two investigators (JRG and QYW) independently selected researches, retrieved pertinent information, and evaluated the possibility of bias. Discrepancies were resolved by consensus with the review team (JRG, QYW, and XX).

Inclusion criteria for this meta-analysis were pre-determined: (1) only chronic randomized controlled trials with at least one type of exercise for depression; (2) individuals (18 years of age or older) diagnosed with MDD based on accepted or established diagnostic criteria (e.g., Beck Depression Inventory-II ≥13) (25); (3) a control group receiving usual care or placebo (e.g., stretching); (4) studies reporting global cognition or domain-specific measures (e.g., executive function, attention); (5) articles in English language journals. We excluded studies combining multiple treatments (e.g., exercise plus cognitive therapy), those with participants having severe health conditions (e.g., Parkinson’s), and studies with intervention durations <4 weeks.

### 2.2 Data extraction and coding strategy

General study characteristics was extracted by three reviewers (JRG, QYW, XX), including participant characteristic, exercise protocol, control group, and main results (i.e., mean and standard deviation (SD) for calculating effect sizes). The diagnostic criteria for MDD are determined by a physician, while cognitive function is assessed by psychologists using standardized instruments and tasks. (see Supplementary Table 5).

To clarify the posterior data synthesis process, we defined data coding in four hierarchical levels. First, interventions were coded as “Exercise” or “Control”; second, as “Aerobic Training,” “Resistance Training,” or “Control.” At the third level, interventions were categorized by exercise type: “Cycling,” “Mixed aerobic exercises,” “Strength,” “Tai-chi/Qigong,” “Walking/Jogging,” and “Placebo” (control). Finally, interventions were classified based on their energy expenditure (Metabolic Equivalent of Task, MET), expressed as METs-min per week, determined by the product of exercise duration, frequency, and intensity (26), We then grouped interventions into four categories based on METs-min per week: 0 (control), 500, 750, and 1,000 METs-min. This classification was made to improve network connectivity, necessary for the network meta-analysis (27).

### 2.3 Three-level meta-analysis

This method was applied to address multiple effect sizes and their non-independence. It accounts for sampling variance (Level 1), within-study variance (Level 2), and between-study variance (Level 3), using restricted maximum likelihood (REML) estimation to calculate standardized mean difference (SMD) (e.g., Hedges’ g) and 95% confidence intervals (CI) for the effect of exercise on CF. The meta-analysis synthesized CF assessments, conducted subgroup analyses on CF subdomains and study/sample characteristics, and explored significant factors through regression analysis.

### 2.4 Bayesian model-based network meta-analysis

A Bayesian model-based network meta-analysis (MBNMA) (28) was employed to provide an overview of the dose–response relationship between cognitive performance and exercise. No violations of key assumptions for network meta-analysis (connectivity, consistency, and transitivity) were found (see Supplementary File 2). Effect sizes were standardized as Hedges’ g (29), with posterior medians and 95% credible intervals (CrI) to assess certainty (30).

We used non-linear models to describe the data and illustrated how various forms and dosages of physical activity affected CF, including Emax, restricted cubic splines and non-parametric monotonically up functions (31). Fit indices (32) and deviance plots were used to compare models (see Supplementary Figures 6, 7), with restricted cubic splines providing the best fit for dose–response associations (see Supplementary Figure 5). Based on the optimal fit model (see Supplementary Table 2) and biological plausibility (31), three knots were positioned at the 10th, 50th, and 90th percentiles of exercise dose (33). To ensure interpretability and adequate network connectivity, exercise doses were grouped into four METs-min categories (0, 500, 750, and 1,000 METs-min per week). These thresholds were derived based on WHO recommendations for physical activity (500–1,000 METs-min/week as the optimal range for health benefits) and the dose distribution across included studies. This categorization allowed for both clinical relevance and statistical stability in the MBNMA. The “optimal dose” of physical activity for the maximal possible cognitive benefit was estimated using beta coefficients from restricted cubic splines. This data was then used to order the examined treatments from worst to best according to their likelihood of improving CF.

The Minimum Clinically Important Difference (MCID) (34) was defined as 0.5 SD, following established conventions in neuropsychological and clinical trials research. A change of this magnitude has been shown to correspond to a perceptible and clinically meaningful improvement in cognitive functioning among individuals with MDD. MCID was used to determine the exercise dose for clinically significant cognitive improvements in MDD, assessed via Montreal Cognitive Assessment (MoCA) (35) and Mini-Mental State Examination (MMSE) (36), with effect sizes compared to a standardized MCID of 0.5 SD (37).

### 2.5 Statistical analysis

Both three-level meta-analysis and Bayesian meta-analyses were conducted in R 4.4.1 (38). We used the ‘MBNMA dose’ package (39) to perform Bayesian dose–response MBNMA models; the ‘metaphor’ package to conduct a three-level meta-analysis (40); the ‘brms’ package to perform regression with moderator factors (41) and the ‘ggplot2’ package for plotting and visualization (42).

### 2.6 Risk of bias and quality assessment

The studies were evaluated separately by three reviewers (JRG, XX, and YFZ) using the PEDro scale (43) for study quality and the Cochrane Risk of Bias Tool (44) for randomized trials. Study quality was classified as high, moderate and poor. Disagreements were resolved by discussion. In order to assess their influence on the total dose–response estimates, a sensitivity analysis was also carried out, eliminating studies with a high risk of bias.

## 3 Results

### 3.1 Study selection

**Figure 1:**
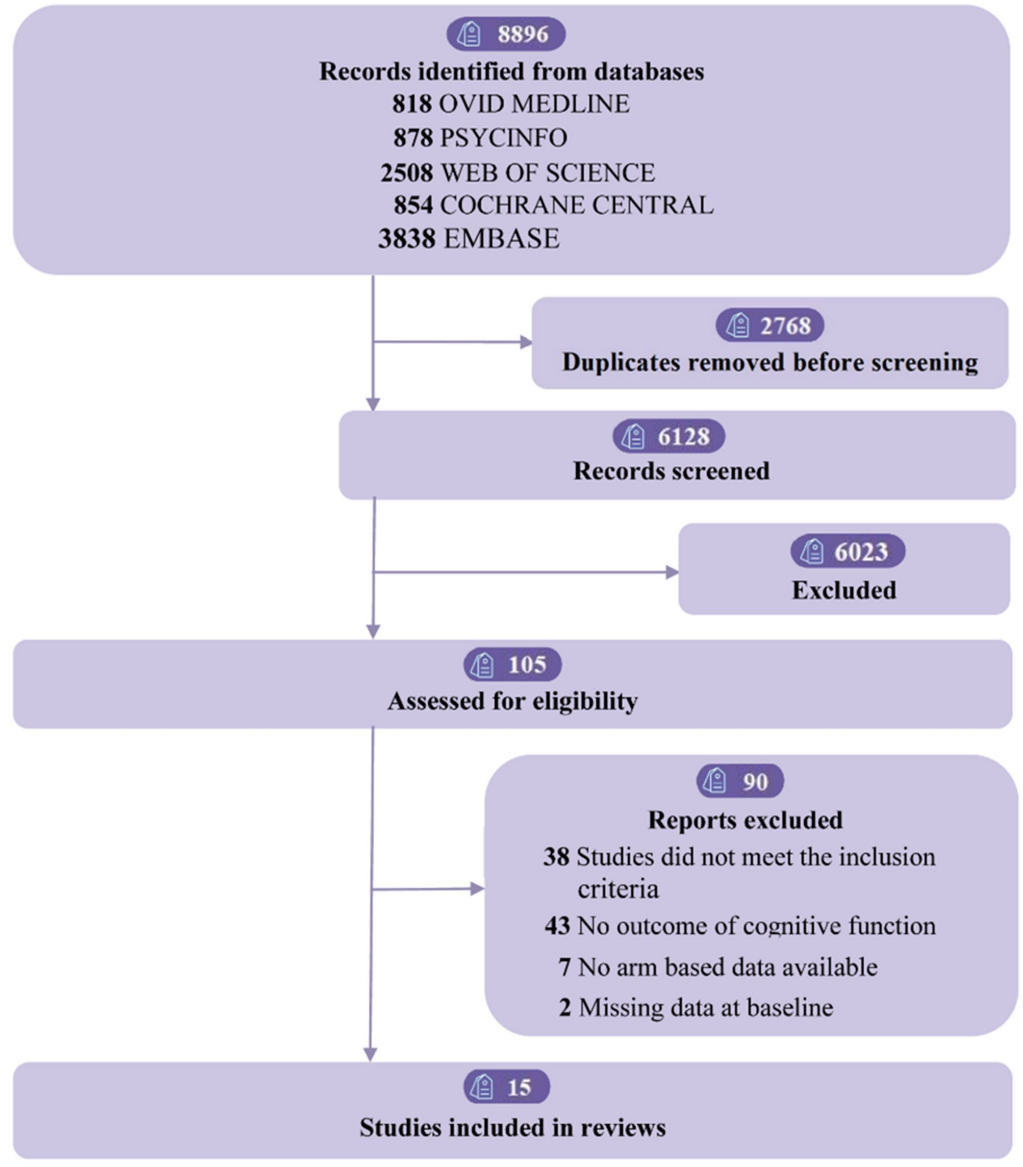
Flow of studies through review.

The study selection procedure is depicted in the PRISMA flow diagram (Figure 1). We retrieved 8,896 studies, and after removing duplicates, 6,128 remained. After two reviewers independently vetted abstracts and titles, 105 pertinent studies were found. Following the application of inclusion/exclusion criteria, the meta-analysis contained 15 studies (43–59), with 36 arms and 1,196 participants. Supplementary Table 5 details the references and characteristics of these studies. Of the participants, 438 (36.6%) were female, with an average age of 42.3 years (SD = 14.5). A total of 337 (28.2%) were overweight, and 630 (52.7%) had mild to moderate depression. The treatment-level and agent-level network diagram is shown in Supplementary Figures 1, 2.

### 3.2 Global cognitive function

This study demonstrated that different types of exercise (135 effect sizes, k = 15) had a small but significant effect on overall cognitive function [(SMD, 0.329; 95% CI, 0.25 to 0.41); standard error (SE) = 0.04]. The likelihood ratio tests (LRTs) showed nonsignificant variance in effects within studies at level 2 (LRT = 0, *p* > 0.05), while showing significant variance between studies at level 3 (LRT = 6.75, *p* = 0.0094). The statistical heterogeneity in the aggregated effect magnitude was minimal (I^2^ = 17.30%). A *p*-value below 0.05 indicates significant heterogeneity, necessitating a moderator analysis to explore its origins.

### 3.3 Moderator analyses

Table 1 presents subgroup analyses based on gender, age, BMI, depression severity, and CF subdomains, identifying key moderators of CF. Exercise had a significant impact, especially in females (SMD, 0.41; 95% CI, 0.22–0.60) and young adults (SMD, 0.32; 95% CI, 0.14–0.51), with minimal effects in middle-aged (SMD, 0.09; 95% CI, −0.21–0.38) and older adults (SMD, −0.13; 95% CI, −0.68–0.41). BMI showed no significant role, whether normal (SMD, 0.09; 95% CI, −0.28–0.45) or overweight (SMD, 0.36; 95% CI, −0.06–0.79). Exercise had the largest effect in individuals with mild to moderate depression (SMD, 0.26; 95% CI, 0.08–0.4). However, it did not significantly improve cognitive subdomains, including executive function (SMD, −0.07; 95% CI, −0.53–0.40), memory (SMD, 0.05; 95% CI, −0.38–0.48; *p* = 0.81), attention (SMD, −0.01; 95% CI, −0.45–0.44), and processing speed (SMD, −0.10; 95% CI, −0.55–0.37).

**Table 1:** Moderator analyses of exercise on cognition.

Subgroup analysis	k	Hedges’g (95% CI)	*p*-value	Test of moderation
Proportion of males	15			*F*(2,14) = 1.60, *p* = 0.24
>50%	8	−0.16 (−0.40,0.09)	0.19	
≤50%	8	**0.41** **(0.22,0.60)**	**<0.001**	
Unclear	1	0.30 (−0.46,1.06)	0.41	
Mean age	15			*F*(2,14) = 0.40, *p* = 0.68
Young adults (18–44 yrs)	9	**0.32 (0.14,0.51)**	**0.002**	
Middle adults (45–64 yrs)	4	0.09 (−0.21,0.38)	0.56	
Old adults (≥65 yrs)	3	−0.13 (−0.68,0.41)	0.61	
BMI	15			*F*(2,14) = 1.77, *p* = 0.21
Healthy	1	0.09 (−0.28,0.45)	0.62	
Overweight	5	0.36 (−0.06,0.79)	0.09	
Unclear	11	0.22 (−0.17,0.62)	0.25	
Depression severity	15			*F*(2,14) = 1.06, *p* = 0.37
Mild to moderate	8	**0.26 (0.08,0.44)**	**0.007**	
Moderate to severe	2	0.01 (−0.66,0.77)	0.87	
Unclear	7	0.19 (−0.09,0.46)	0.17	
Cognitive sub-domains	13			*F*(4,14) = 0.124, *p* = 0.971
Executive function	12	−0.07 (−0.53,0.40)	0.76	
Memory	9	0.05 (−0.38,0.48)	0.81	
Attention	4	−0.01 (−0.45,0.44)	0.98	
Processing speed	5	−0.10 (−0.55,0.37)	0.67	

k, number of included studies; CI, confidence interval; yrs, years. Bold values indicate statistically significant effects (*p* < 0.05).

**Figure 2:**
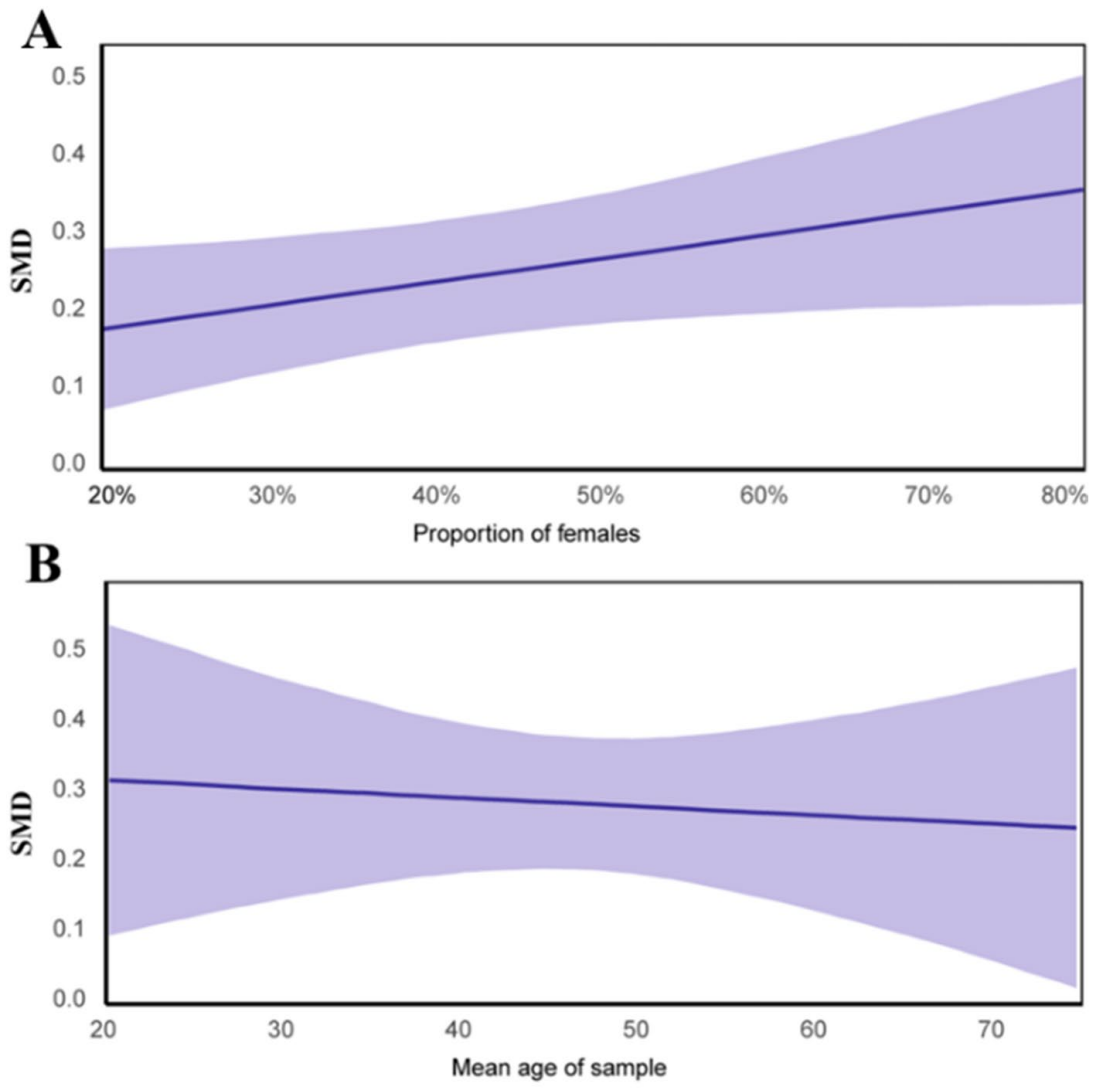
Regression analysis between effects of cognitive function and moderators (sex and age). **(A)** Regression between proportion of females and cognitive effect size; **(B)** Regression between mean age and cognitive effect size.

Figure 2 shows the linear regression between cognitive function and the two key moderators: gender and age. Depression severity was excluded to maintain objectivity. The results indicate that younger participants and those with a higher proportion of females experience more significant cognitive improvements from exercise. In studies with participants aged 18–44 and a female proportion >50%, the fitted dose–response relationship shows MCID values of 501 METs-min/week and 761 METs-min/week, respectively (see Supplementary Figures 8, 9).

### 3.4 Dose–response relationships

**Figure 3:**
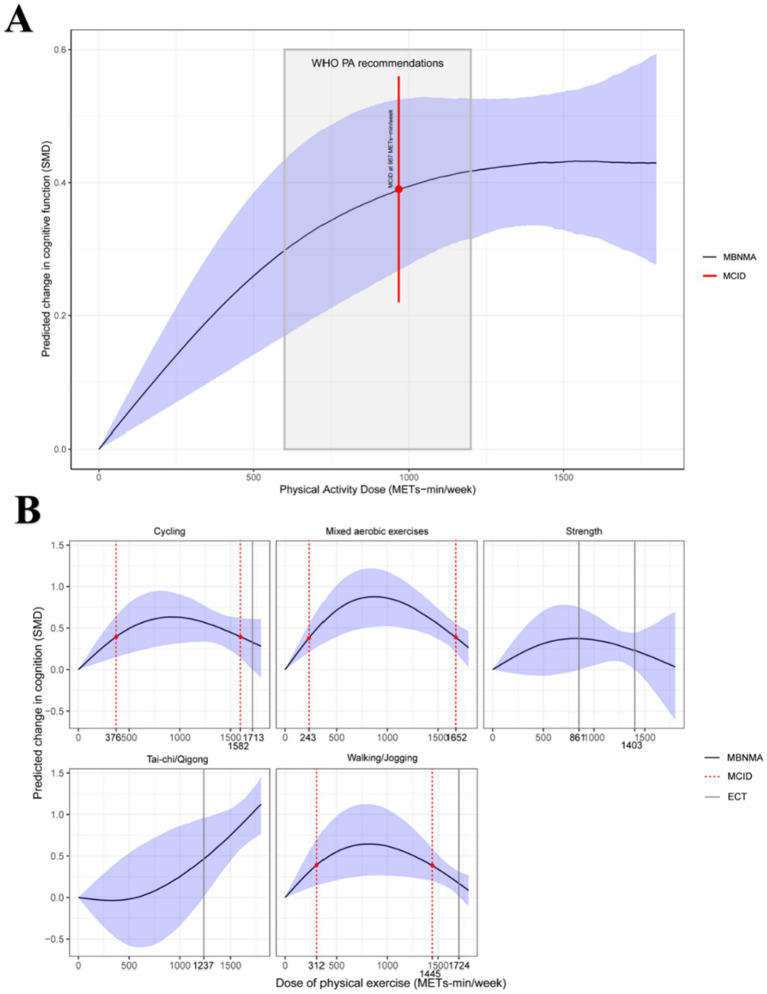
Dose–response relationship between overall physical activity and different types of physical activity on cognitive function in adults with MDD. MBNMA, Model-based network meta-analysis; MCID, Minimum clinically important difference; ECT, Effective critical threshold. **(A)** Overall dose–response relationship between physical activity and cognitive function; **(B)** Dose–response relationships by exercise type.

Figure 3 illustrates the estimated dose–response relationship between physical activity dose and cognitive improvement in adults with MDD.

Panel A shows the nonlinear dose–response relationship between exercise and CF. CF improves significantly beyond 600 METs-min (WHO’s lower bound), with a slight plateau after 1,200 METs-min (WHO’s upper bound) (57). The combined effect size, based on the minimal clinically important difference (MCID), is moderate (SMD = 0.39), with values of 0.29 [95% CrI, 0.17–0.43] at 600 METs-min and 0.42 [95% CrI, 0.32–0.53] at 1200 METs-min. The minimum exercise dose to achieve this effect is approximately 967 METs-min/week (SMD, 0.39; 95% CI, 0.27–0.53). Predicted effects for the lower and upper WHO activity levels are shown in Supplementary Table 4. Panel A indicates that cognitive benefits increase rapidly up to approximately 967 METs-min/week, after which the curve plateaus. This threshold corresponds to the minimum effective dose reaching the minimum clinically important difference (MCID, 0.5 SD), which lies within the WHO-recommended range (500–1,000 METs-min/week).

Figure 3 illustrates the dose–response relationship between exercise types and CF in adults with MDD. Cycling, Mixed aerobic exercises, and Walking/Jogging show inverted U-shaped patterns, with peak effect sizes at 914 METs-min (SMD, 0.63; 95% CrI, 0.31–0.93), 864 METs-min (SMD, 0.89; 95% CrI, 0.52–1.23), and 810 METs-min (SMD, 0.64; 95% CrI, 0.26–1.12), respectively. The MCID for these exercises ranges from 376 to 1,582 METs-min/week, 243–1,652 METs-min/week, and 312–1,445 METs-min/week. At doses of 1713 METs-min/week (SMD, 0.33; 95% CrI, 0–0.61) and 1724 METs-min/week (SMD, 0.17; 95% CrI, 0–0.31), Cycling and Walking/Jogging no longer show significant effects, roughly three times the lower limit of the WHO’s recommended exercise dose (58). The Strength exercise dose–response curve follows a whale-like shape, with significant effects only within 861–1,403 METs-min/week and no MCID or maximum dose. Tai-chi/Qigong exhibits a U-shaped pattern, but its wide CI limits significant effects to 1,237 METs-min/week (SMD, 0.47; 95% CrI, 0–0.96).

Panel B presents the dose–response patterns by exercise type. Tai Chi/Qigong demonstrated a nearly linear improvement across the exercise range, whereas aerobic modalities (cycling, walking/jogging, and mixed aerobic exercises) exhibited an inverted-U pattern, with optimal effects observed between 750 and 1,200 METs-min/week. Resistance training showed smaller but consistent improvements. These findings highlight both a global optimal dose and exercise-specific variations in cognitive response.

Supplementary Table 3 compares the effects of different exercises at various doses. Tai-chi/Qigong (1,000 METs-min/week) shows the most favorable effect, followed by Mixed aerobic exercise (500 METs-min/week). Table 2 offers practical exercise recommendations based on these findings.

**Table 2:** Exercise recommendations to improve cognitive function for adults with major depressive disorder.

Type of physical activity	MCID (MET-min/week)	Significant responses (MET-min/week)	Intensity	Energy expenditure^1^ (METs-min)	Recommended accumulation (min/week)	
					Minimum	Optimal
Cycling	376–1,582		Moderate	3.8 (code 15400)	~95	~230
			Vigorous	8.5 (code 01008)	~45	~115
Mixed aerobic exercises	243–1,652		Moderate	4.0 (code 15140, 15232)	~60	~215
			Vigorous	8.0 (code 12025, 15533)	~30	~105
Walking/Jogging	312–1,445		Moderate	4.0 (code 17100, 17133)	~75	~200
			Vigorous	7.8 (code 17012)	~40	~100
Strength		861–1,403	Moderate	4.0 (code 15440)	~215	~215
			Vigorous	8.3 (code 15560)	~105	~105
Tai-chi/Qigong		1,237	Moderate	4.0 (code 15170)	~305	
			Vigorous	8.3 (code 17231)	~150	

^1^Intensity coding was extracted from the compendium of physical activity (70).

Across included studies, cognitive domains most frequently assessed were executive function, attention, and memory. Subgroup analyses suggested slightly stronger effects on executive function and attention, consistent with previous evidence linking exercise to fronto-executive network enhancement in MDD.

### 3.5 Risk of bias and quality assessment

Of the studies, six were rated low risk, five moderate risk, and four high risk (see Supplementary Figure 11). The average quality score was 6.25 (see Supplementary Table 6). Sensitivity analyses, excluding high-risk and unclear studies, confirmed an inverted U-shaped dose–response relationship, with an optimal dose of 923 METs-min/week (see Supplementary Figure 10).

## 4 Discussion

### 4.1 Nonlinear associations between exercise and cognitive function

This study reveals several clinically significant findings. First, physical activity interventions improve cognitive function (CF) in adults with MDD. Second, our results demonstrate a distinct non-linear connection between exercise and CF, with impact values ranging from minor to moderate. The minimum clinically effective dose is 967 METs-min per week, equivalent to 250 min of moderate-intensity exercise or 125 min of vigorous exercise, consistent with WHO guidelines (19). This suggests that maintaining a tolerable level of weekly physical activity is beneficial for CF, offering a reference for experts in developing exercise plans for MDD patients, many of whom may lack motivation for physical activity. However, cognitive improvements plateau once the dose exceeds the WHO-recommended 1,200 METs-min/week. Notably, the dose–response relationship between exercise and CF mirrors that observed for alleviating depressive symptoms (59). Specifically, before reaching the WHO-recommended dose, higher exercise levels correlate with better outcomes, which could guide research on increasing exercise doses for patients whose CF remains impaired despite improvements in depression (60).

### 4.2 Exercise types and dose–response relationships in cognitive function

Most exercise types in our study effectively improve CF, with identifiable dose–response patterns. Notably, Tai Chi/Qigong emerged as the most effective for enhancing CF in MDD patients, though a certain intensity is required, and further validation is needed. For other exercise types, dose–response relationships generally follow an inverted U-shape, consistent with a similar research in older adults (61), though our effect sizes may be more pronounced. This relationship likely varies by exercise type, reflecting different mechanisms that induce cognitive changes. Tai Chi/Qigong’s effectiveness may stem from its inherent integration of physical exercise with cognitive therapy. Beyond physiological adaptations, the superior efficacy of Tai Chi and Qigong observed in this study may be mediated by their dual emphasis on physical exertion and mental focus. Previous research indicates that mind–body interventions significantly reduce sympathetic nervous system activity and cortisol levels more effectively than aerobic exercise alone (62). These modalities require sustained attention and motor planning, which may directly engage the prefrontal cortex and hippocampus—regions often compromised in MDD. Consequently, the integration of mindfulness with movement appears to offer a synergistic benefit for executive control and working memory that pure resistance or aerobic training may not fully replicate. However, the minimum threshold dose for this intervention is 1,237 METs-min per week, which requires a considerable time and physical commitment from most patients. Therefore, patients might consider more accessible exercises like cycling, mixed aerobic exercises, and walking, which also produce significant effects. Their minimum clinically important differences are 376, 243, and 312 METs-min per week, respectively.

Our study categorizes exercises into two main types based on training characteristics: aerobic exercise and resistance training. Adults with MDD can improve their CF with aerobic exercise (63), which aligns with our findings. Other meta-analyses also show its significant impact on cognitive function in conditions like schizophrenia and dementia (64), making it the most common and effective intervention. The mechanisms behind its effects on cognitive performance require further exploration from a neuropsychological perspective (65). In contrast, resistance training appears to be a higher-threshold and less effective intervention for MDD patients. We could not identify its optimal dose or MCID, and its effectiveness is limited to a range of 861 to 1,403 METs-min per week. However, some studies suggest that even low doses of resistance training can induce clinically significant cognitive changes and may offer superior overall cognitive improvement (66). This discrepancy could stem from their focus on older populations.

### 4.3 The influence of moderators’ factors on cognitive function

Gender and age were identified as significant moderators of CF in adults with MDD. Evidence shows that individuals aged 18–44 years are particularly affected, with a weekly beneficial dose of at least 501 METs-min. The moderating effect is more pronounced in studies with over 50% female participants.

The moderation analysis revealed stronger cognitive benefits in younger adults (18–44 years) and females. Biological mechanisms likely underpin the age-related findings; younger adults typically exhibit greater neuroplasticity and higher baseline levels of Brain-Derived Neurotrophic Factor (BDNF) in response to physical stress compared to older populations (67). Regarding gender, the stronger response observed in women may be linked to the interaction between sex hormones and the dopaminergic system. Estrogen has been shown to enhance synaptic plasticity and may potentiate the neurotrophic effects of exercise (68). Furthermore, sociodemographic data suggests that women with MDD may exhibit different baseline physical activity behaviors and adherence rates compared to men, potentially influencing the net effectiveness of the intervention.

### 4.4 Strengths, limitations and future directions

Mechanistically, the cognitive enhancement observed in MDD through exercise may involve increased neuroplasticity, elevated brain-derived neurotrophic factor (BDNF) levels, reduced systemic inflammation, and improved stress regulation via the hypothalamic–pituitary–adrenal (HPA) axis. Exercise can also enhance prefrontal and hippocampal activity, regions crucial for attention, memory, and emotional regulation. While part of the cognitive improvement may result from alleviation of depressive symptoms, emerging evidence suggests that exercise-induced neurobiological changes independently contribute to cognitive restoration.

Our research has a number of advantages. First, we applied a three-level meta-analysis alongside traditional network meta-analysis to address test result merging and manage heterogeneity within and between studies. Second, we used the latest Bayesian models to estimate exercise doses, providing more precise insights, particularly for developing exercise prescriptions. Finally, our findings align with the WHO’s Global Action Plan on Physical Activity (GAPPA) target for 2030, promoting “more active people for a healthier world.” Precise exercise dosing allows office workers and students to achieve similar benefits to continuous exercise by accruing brief bursts of exercise over the course of the week, known as “exercise snacks” (69). Nevertheless, this study has several limitations. First, experimental biases may have arisen from human factors, as both staff and participants could infer the study’s objectives. Exercise interventions, unlike pharmaceutical ones, are especially challenging to double-blind. Future studies should ensure the research hypothesis remains unknown to all parties, ideally with third-party supervision. Second, moderating factors like gender and age were assessed at the study level, not the individual level, which may explain some heterogeneity. To more accurately evaluate these factors, future reviews ought to take individual participant meta-analyses into account.

## 5 Conclusion

This research delineates the therapeutic effect of exercise on CF in individuals with MDD, identifying dose–response connections between exercise kinds and cognitive outcomes. Aerobic exercise requires a minimum of 60 min per week to improve CF, a feasible amount for all age groups, while strength training needs about 210 min per week for similar benefits. These findings offer valuable exercise-related recommendations to help address the global challenge of depression.

## Data Availability

The original contributions presented in the study are included in the article/Supplementary material, further inquiries can be directed to the corresponding authors.
