# Supplementary material for: Optimal dose and type of exercise to improve cognitive function in adults with major depressive disorder: a systematic review and Bayesian model-based network meta-analysis
Source: Front Public Health. 2025 Dec 19;13:1662778. doi: 10.3389/fpubh.2025.1662778 (PMC12757253; doi:10.3389/fpubh.2025.1662778)
Supplement: Supplementary file 1 [file Supplementary_file_1.docx]

# Optimal dose and type of exercise to improve cognitive function in adults with major depressive disorder：

# A systematic review and Bayesian model-based network meta-analysis

**Table of Contents**

[Supplementary file 1: Search strategy 3](#_TOC_250009)

[Supplementary file 2: Key assumption of Network Meta-Analysis 8](#_TOC_250008)

[Connectivity *8*](#_TOC_250007)

Supplementary Figure 1. Treatment-level network 8

Supplementary Figure 2. Agent-level network 9

[Consistency *9*](#_TOC_250006)

Supplementary Table 1. Consistent and UME models fit comparison 9

[Transitivity *10*](#_TOC_250005)

Supplementary Figure 3. Node-splitting analysis (forest plot) 10

Supplementary Figure 4. Node-splitting analysis (density plot) 11

[Supplementary *file 3:* Non-linear functions and models fit comparison *12*](#_TOC_250004)

Supplementary Figure 5. “Split” NMA of overall exercise 12

Supplementary Table 2. Model fit comparison 12

Supplementary Figure 6. Deviance plot at overall exercise level 13

Supplementary Figure 7. Deviance plots at treatment-level 14

[Supplementary *file 4:* League table, predicted responses *15*](#_TOC_250003)

[Supplementary Table 3. League table for ranking of effectiveness 15](#_TOC_250002)

[Supplementary Table 4. Predicted responses at 600,1200 METs per week 16](#_TOC_250001)

Supplementary *file 5:* Characteristics of included studies *17*

Supplementary Table 5. Overview of selected studies 17

Supplementary *file 6:* Quality assessment of included studies *20*

[Supplementary Table 6. Quality Assessment Sheet 20](#_TOC_250000)

Supplementary file 7: Moderate analysis 21

Supplementary Figure 8. Dose-response curve (Sex) 21

Supplementary Figure 9. Dose-response curve (Age) 22

**Supplementary *file 8:* Sensitivity analysis *23***

**Supplementary Figure 10.** Dose-response curve only including studies with low risk of bias ***23***

**Supplementary *file 9:* Risk of bias  *24***

**Supplementary Figure 11.** Cochrane Risk of Bias Tool  ***24***

**References *25***

## Supplementary file 1:Search strategy

| **Ovid Medline** | 1. exp Exercise/ |
| --- | --- |
| **(2024/9/20)** | 2. exp Exercise Therapy/ |
|  | 3. exp "Physical Education and Training"/ |
|  | 4. Physical Fitness/ |
|  | 5. Physical Exertion/ |
|  | 6. exp Walking/ |
|  | 7. Running/ or Jogging/ |
|  | 8. Swimming/ or Yoga/ or Qigong/ or Tai ji/ |
|  | 9. (cycling or bicycling or yoga or "tai-chi" or "tai chi" or "tai ji" or |
|  | qigong or "qi gong").tw. |
|  | 10. (exercise$ or exercising).tw. |
|  | 11. (physical adj3 (education or training)).tw. |
|  | 12. 1 or 2 or 3 or 4 or 5 or 6 or 7 or 8 or 9 or 10 or 11 |
|  | 13. Depression/ |
|  | 14. exp Depressive Disorder/ |
|  | 15. 13 or 14 |
|  | 16. Cognition/ |
|  | 17. Comprehension/ |
|  | 18. Memory/ |
|  | 19. Metacognition/ |
|  | 20. Perception/ |
|  | 21. Executive Function/ |
|  | 22. Attention/ |
|  | 23. cognition*.tw. |
|  | 24. cognitive*.tw. |
|  | 25. neurocognit*.tw. |
|  | 26. comprehension.tw. |
|  | 27. memory.tw. |
|  | 28. perception.tw. |
|  | 29. attention*.tw. |
|  | 30. 16 or 17 or 18 or 19 or 20 or 21 or 22 or 23 or 24 or 25 or 26 or 27 |
|  | or 28 or 29 |
|  | 31. randomized controlled trial.pt. |
|  | 32. controlled clinical trial.pt. |
|  | 33. randomly.ab. |
|  | 34. trial.ab. |
|  | 35. groups.ab. |
|  | 36. (control$ adj3 (trial$ or study or studies)).tw. |
|  | 37. randomi#ed.ab. |
|  | 38. placebo$.ab. |
|  | 39. 31 or 32 or 33 or 34 or 35 or 36 or 37 or 38 |
|  | 40. 12 and 15 and 30 and 39 |

|  | Results=818 |
| --- | --- |
| **PSYCINFO** | TI ( Depress* OR Depression OR Depressive OR Depressed ) OR SU |
| **(2024/9/20)** | ( Depress* OR Depression OR Depressive OR Depressed ) OR DE |
|  | ( Depress* OR Depression OR Depressive OR Depressed ) OR AB |
|  | ( Depress* OR Depression OR Depressive OR Depressed ) OR KW |
|  | ( Depress* OR Depression OR Depressive OR Depressed ) AND TI |
|  | ( Aerobic Exercise OR Aerobic Exercises OR Exercise OR exercis* OR |
|  | aerobic OR physical activit* OR physical endurance OR Fitness OR |
|  | walking ) OR SU ( Aerobic Exercise OR Aerobic Exercises OR |
|  | Exercise OR exercis* OR aerobic OR physical activit* OR physical |
|  | endurance OR Fitness OR walking ) OR DE ( Aerobic Exercise OR |
|  | Aerobic Exercises OR Exercise OR exercis* OR aerobic OR physical |
|  | activit* OR physical endurance OR Fitness OR walking ) OR AB |
|  | ( Aerobic Exercise OR Aerobic Exercises OR Exercise OR exercis* OR |
|  | aerobic OR physical activit* OR physical endurance OR Fitness OR |
|  | walking ) OR KW ( Aerobic Exercise OR Aerobic Exercises OR |
|  | Exercise OR exercis* OR aerobic OR physical activit* OR physical |
|  | endurance OR Fitness OR walking ) AND TI ( Cognition OR cogniti* |
|  | OR neuro* OR neurocogniti* OR Metacognitive Control OR |
|  | Metacognitive Controls OR attention OR processing speed OR Memory |
|  | OR executive function* OR executive control* OR executive process* |
|  | OR inhibition OR inhibitory control OR updating OR working memory |
|  | OR shifting OR set shifting OR switching OR task switching OR |
|  | cognitive flexibility OR planning OR problem solving OR |
|  | problem-solving OR reasoning ) OR SU ( Cognition OR cogniti* OR |
|  | neuro* OR neurocogniti* OR Metacognitive Control OR Metacognitive |
|  | Controls OR attention OR processing speed OR Memory OR executive |
|  | function* OR executive control* OR executive process* OR inhibition |
|  | OR inhibitory control OR updating OR working memory OR shifting |
|  | OR set shifting OR switching OR task switching OR cognitive |
|  | flexibility OR planning OR problem solving OR problem-solving OR |
|  | reasoning ) OR DE ( Cognition OR cogniti* OR neuro* OR |
|  | neurocogniti* OR Metacognitive Control OR Metacognitive Controls |
|  | OR attention OR processing speed OR Memory OR executive function* |
|  | OR executive control* OR executive process* OR inhibition OR |
|  | inhibitory control OR updating OR working memory OR shifting OR |
|  | set shifting OR switching OR task switching OR cognitive flexibility |
|  | OR planning OR problem solving OR problem-solving OR reasoning ) |
|  | OR AB ( Cognition OR cogniti* OR neuro* OR neurocogniti* OR |
|  | Metacognitive Control OR Metacognitive Controls OR attention OR |
|  | processing speed OR Memory OR executive function* OR executive |
|  | control* OR executive process* OR inhibition OR inhibitory control |
|  | OR updating OR working memory OR shifting OR set shifting OR |

|  | switching OR task switching OR cognitive flexibility OR planning OR problem solving OR problem-solving OR reasoning ) OR KW ( Cognition OR cogniti* OR neuro* OR neurocogniti* OR Metacognitive Control OR Metacognitive Controls OR attention OR processing speed OR Memory OR executive function* OR executive control* OR executive process* OR inhibition OR inhibitory control OR updating OR working memory OR shifting OR set shifting OR switching OR task switching OR cognitive flexibility OR planning OR problem solving OR problem-solving OR reasoning ) AND TI ( Randomized Controlled Trial OR Randomized OR randomised OR random OR randomly OR randomness OR Randomization OR control clinical trial OR controlled clinical trial OR randomized clinical trial OR Randomized Controlled study OR randomized clinical study ) OR SU ( Randomized Controlled Trial OR Randomized OR randomised OR random OR randomly OR randomness OR Randomization OR control clinical trial OR controlled clinical trial OR randomized clinical trial OR Randomized Controlled study OR randomized clinical study ) OR DE ( Randomized Controlled Trial OR Randomized OR randomised OR random OR randomly OR randomness OR Randomization OR control clinical trial OR controlled clinical trial OR randomized clinical trial OR Randomized Controlled study OR randomized clinical study ) OR AB ( Randomized Controlled Trial OR Randomized OR randomised OR random OR randomly OR randomness OR Randomization OR control clinical trial OR controlled clinical trial OR randomized clinical trial OR Randomized Controlled study OR randomized clinical study ) OR KW ( Randomized Controlled Trial OR Randomized OR randomised OR random OR randomly OR randomness OR Randomization OR control clinical trial OR controlled clinical trial OR randomized clinical trial OR Randomized Controlled study OR randomized clinical study ) OR PT ( randomized controlled trials or rtc or randomised control trials )  Results=878 |
| --- | --- |
| **Web of science (2024/9/20)** | TI=(Depress* OR Depression OR Depressive OR Depressed) AND TS=(Exercise OR Exercises OR exercis* OR Physical activity OR Physical activities OR physical activit* OR Aerobic OR anaerobic OR multicomponent exercise OR multidisciplinary exercise OR Fitness OR Physical Fitness OR Resistance exercise OR Resistance exercises OR Resistance training OR power training OR Strength Training OR Weight-Lifting OR weightlifting OR weight train OR weight training OR Weight Lifting Strengthening Program OR Strengthening Program OR Weight Bearing Strengthening Program OR Weight-Bearing Strengthening Programs OR Weight Bearing OR muscle strength OR  Sports OR Sport OR Running OR Jogging OR Treadmill training OR |

|  | Walking OR Aquatic Cycling OR Aquatic exercise OR Stretching OR Endurance Training OR Physical Endurance OR High-intensity Interval Training OR High-Intensity* OR HIIT OR Body-building exercises OR Balance training OR Flexibility training) AND TS=(Cognition OR cogniti* OR neuro* OR neurocogniti* OR Metacognitive Control OR Metacognitive Controls OR attention OR processing speed OR Memory OR executive function* OR executive control* OR executive process* OR inhibition OR inhibitory control OR updating OR working memory OR shifting OR set shifting OR switching OR task switching OR cognitive flexibility OR planning OR problem solving OR problem-solving OR reasoning) AND TS=(Randomized Controlled Trial OR Randomized OR randomised OR random OR randomly OR randomness OR Randomization OR control clinical trial OR controlled clinical trial OR randomized clinical trial OR Randomized Controlled study OR randomized clinical study)  Results=2508 |
| --- | --- |
| **Cochrane central (2024/9/20)** | 1. MeSH descriptor: [Exercise] explode all trees 2. MeSH descriptor: [Exercise Therapy] explode all trees 3. MeSH descriptor: [Physical Education and Training] explode all trees 4. MeSH descriptor: [Physical Fitness] this term only 5. MeSH descriptor: [Physical Exertion] this term only 6. MeSH descriptor: [Walking] explode all trees 7. MeSH descriptor: [Running] explode all trees 8. MeSH descriptor: [Swimming] this term only 9. (cycling or bicycling or yoga or “tai-chi” or “tai chi” or “tai ji” or qigong or “qi gong”):ti,ab,kw 10. (exercise* or exercising):ti,ab,kw 11. (physical NEAR/5 (education or training)):ti,ab,kw 12. #1 OR #2 OR #3 OR #4 OR #5 OR #6 OR #7 OR #8 OR #9 OR #10   OR #11   1. MeSH descriptor: [Depressive Disorder] explode all trees 2. MeSH descriptor: [Depression] this term only 3. #13 OR #14 4. MeSH descriptor: [Cognition] explode all trees 5. MeSH descriptor: [Executive Function] explode all trees 6. (Cognition or Cognitions or cogniti* or Cognitive Function or Cognitive Function* or Cognitive Performance or Neurocognitive or Neurocognition or neurocogniti* or Metacognition or Metacognitive Control or Metacognitive Controls or Memory or executive Function or executive functions or executive control or executive controls or cognitive control or cognitive controls or executive process* or Inhibition or inhibitory control or updating or working memory or |

|  | shifting or set shifting or switching or task switching or cognitive flexibility or planning or problem-solving or problem solving or reasoning):ti,ab,kw   1. #16 OR #17 OR #18 2. #12 AND #15 AND #19   Results=854 |
| --- | --- |
| **Embase (2024/9/20)** | ('depression'/exp OR 'depress*':ab,ti OR 'depression':ab,ti OR 'depressive':ab,ti OR 'depressed':ab,ti) AND ('aerobic exercise'/exp OR 'aerobic exercise':ab,ti OR 'aerobic exercises':ab,ti OR 'exercise':ab,ti OR 'exercis*':ab,ti OR 'aerobic':ab,ti OR 'physical activit*':ab,ti OR 'physical endurance':ab,ti OR 'fitness':ab,ti OR 'walking':ab,ti) AND ('cognition'/exp OR 'executive function'/exp OR 'cognition':ab,ti OR 'cogniti*':ab,ti OR 'neuro*':ab,ti OR 'neurocogniti*':ab,ti OR 'metacognitive control':ab,ti OR 'metacognitive controls':ab,ti OR 'attention':ab,ti OR 'processing speed':ab,ti OR 'memory':ab,ti OR 'executive function*':ab,ti OR 'executive control*':ab,ti OR 'executive process*':ab,ti OR 'inhibition':ab,ti OR 'inhibitory control':ab,ti OR 'updating':ab,ti OR 'working memory':ab,ti OR 'shifting':ab,ti OR 'set shifting':ab,ti OR 'switching':ab,ti OR 'task switching':ab,ti OR 'cognitive flexibility':ab,ti OR 'planning':ab,ti OR 'problem solving':ab,ti OR 'problem-solving':ab,ti OR 'reasoning':ab,ti) AND ('randomized controlled trial':ab,ti OR 'randomized':ab,ti OR 'randomised':ab,ti OR 'random':ab,ti OR 'randomly':ab,ti OR 'randomness':ab,ti OR 'randomization':ab,ti OR 'control clinical trial':ab,ti OR 'controlled clinical trial':ab,ti OR 'randomized clinical trial':ab,ti OR 'randomized controlled study':ab,ti OR 'randomized clinical study':ab,ti) Results=3838 |

## Supplementary file 2: Key assumption of Network Meta-Analysis

There are three key assumptions to conduct a Network Meta-Analysis (NMA): (1) network connectivity, (2) consistency in the data, (3) transitivity [[1,2]](https://paperpile.com/c/b8T1z2/kvl9%2BgaquA).

## Connectivity

Connectivity is a key assumption in NMA which if deemed insufficient (i.e., due to lack of direct comparators) can lead to low statistical power and misleading results [[3]](https://paperpile.com/c/b8T1z2/YP82p). In our study, we assessed connectivity of the network at both treatment and agent levels visually and found no evidence of unconnectedness on either network (Supplementary Figure 1 and Supplementary Figure 2).


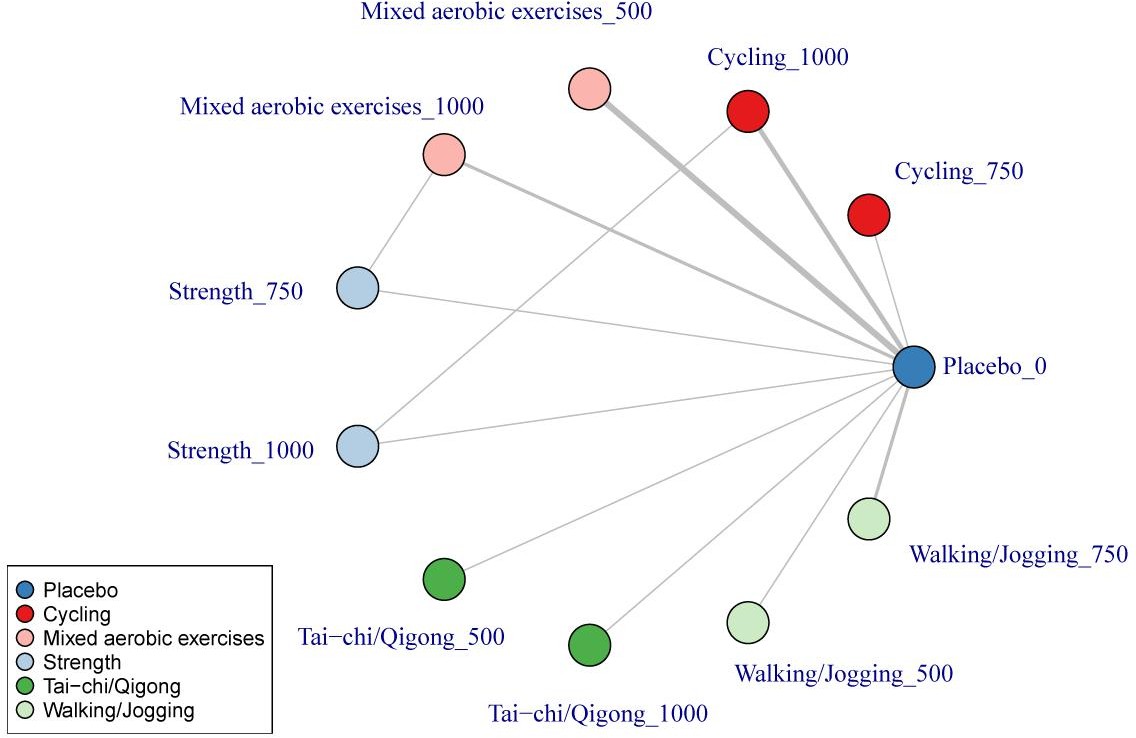


**Supplementary Figure 1**. Treatment-level network.


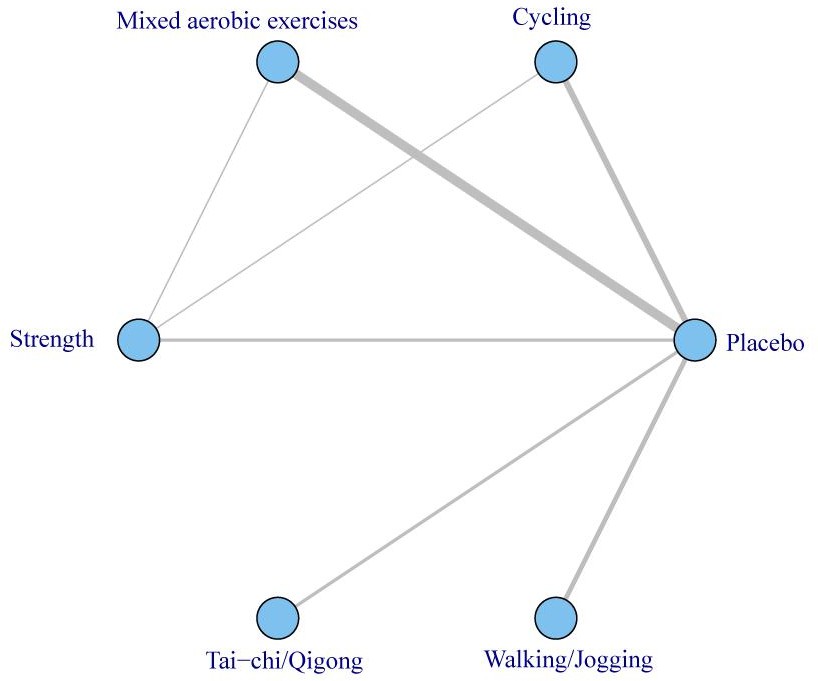


**Supplementary Figure 2.** Agent-level network.

## Consistency

We carried out consistency analysis in the data through the comparison of consistent (i.e., network effect sizes) and unrelated mean effects (UME) models (i.e., pairwise effect sizes) of the network. In practice, we checked whether deviance, the number of estimated parameters in the network, and the Deviance Informative Criterion (DIC) indicators were similar for both models which would indicate a good fit [[4]](https://paperpile.com/c/b8T1z2/jIaJT). Comparison of these parameters indicated good consistency across models (Supplementary Table 1).

**Supplementary Table 1.** Consistent and UME models fit comparison

| **Model** | **pD** | **Residual deviance** | **DIC** | **SD** |
| --- | --- | --- | --- | --- |
| Consistent | 39.3 | 144.1 | 69.1 | 0.145 |
| UME | 39 | 143.6 | 68.3 | 0.146 |

*Note.* pD: Number of estimated parameters; DIC: Deviance Informative Criterion; SD: Standard

Deviation; UME: Unrelated Mean Effects. Scientific literature indicated that the main indicator to assess the model fit is the DIC. As lower DIC, better fit.

## Transitivity


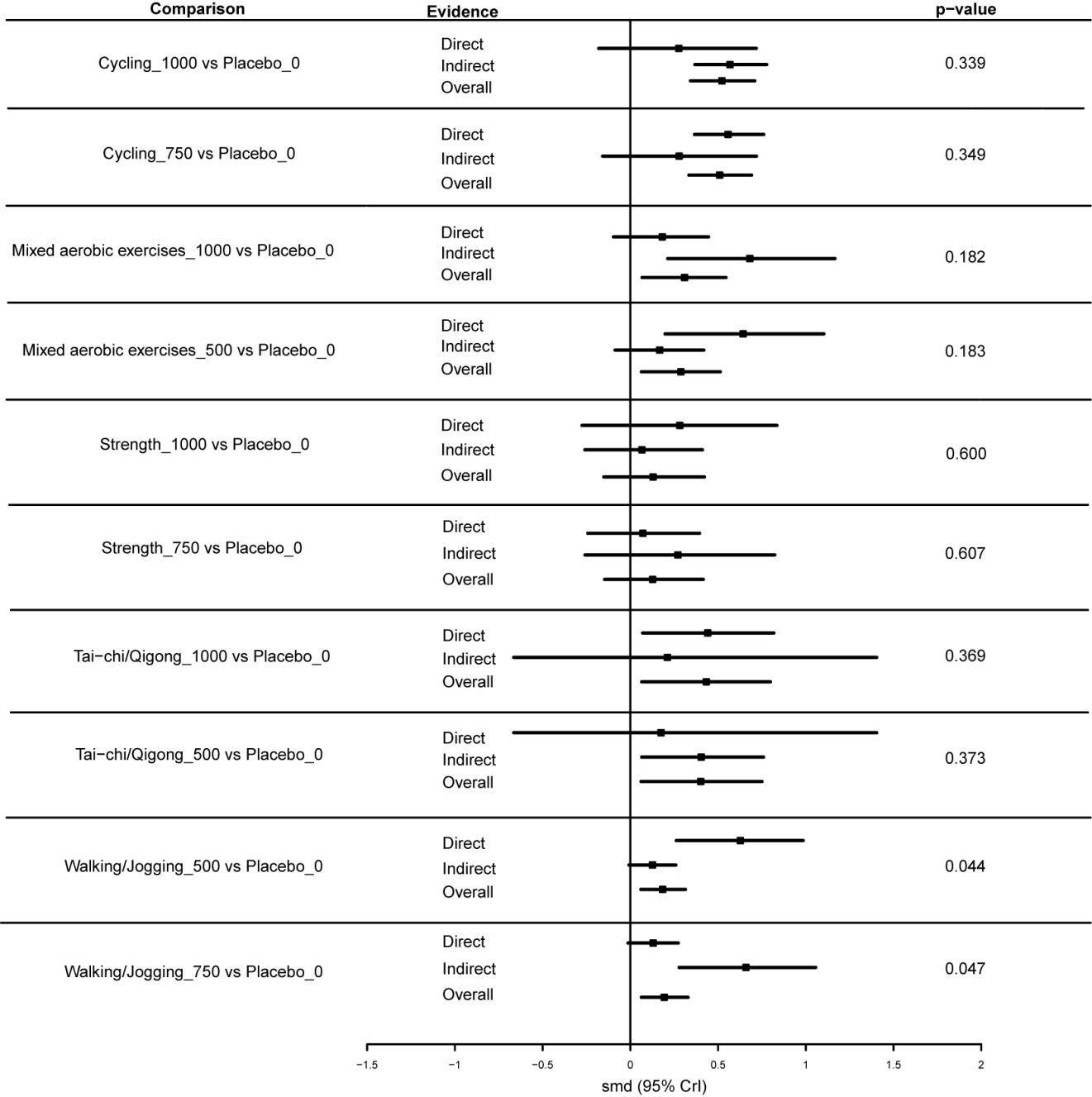
NMAs rest under the assumption of underlying indirect/mixed comparisons, which means the estimates of treatment effects from direct and indirect evidence are in agreement, subject to the usual variation under the random-effects model for meta-analysis [[5]](https://paperpile.com/c/b8T1z2/VBvcg). This assumption is equivalent to heterogeneity in ‘standard’ meta-analysis [[6]](https://paperpile.com/c/b8T1z2/hbtK1). Following previous recommendations [[7]](https://paperpile.com/c/b8T1z2/El6R), transitivity was assessed at the deeper level of the network (i.e., treatment level). We assessed transitivity *via* MBNMA node-splitting approach. This method splits and compares contributions for a particular treatment contrast into direct and indirect evidence [[8]](https://paperpile.com/c/b8T1z2/RQrTH). Similar effects denote good transitivity. Supplementary Figures 3 (point estimates) and 4 (density plots) below present the results for transitivity in this meta-analysis.

**Supplementary Figure 3.** Node-splitting analysis (forest plot)


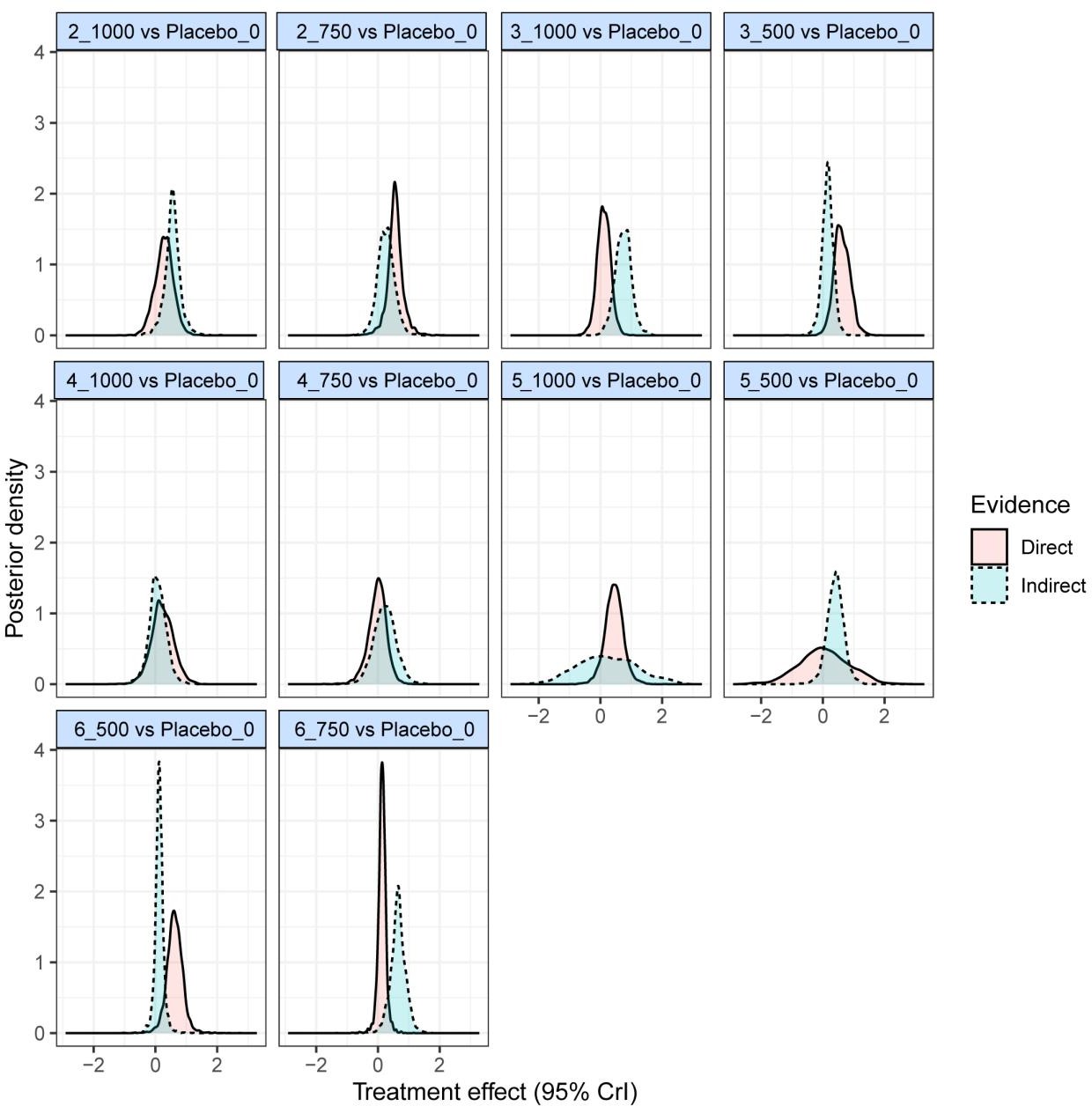


**Supplementary Figure 4.** Node-splitting analysis (density plot). The first value indicates the agent and the second one is the corresponding dose of that agent. 2: Cycling; 3: Mixed aerobic exercises; 4: Strength; 5: Tai-chi/Qigong; 6: Walking/Jogging.

## Supplementary file 3: Non-linear functions and models fit comparison

The different doses of physical activity were meta-analysed as independent and unrelated treatments (i.e., “split” NMA). This step is useful to determine which function fits the data better and should subsequently be used in a Model-Based Network Meta-Analysis (MBNMA) [[9]](https://paperpile.com/c/b8T1z2/q25h). Supplementary Figure 5 show the different responses (Hedges’ g) of each dose for overall physical activity to examine which function is more suitable to this research.


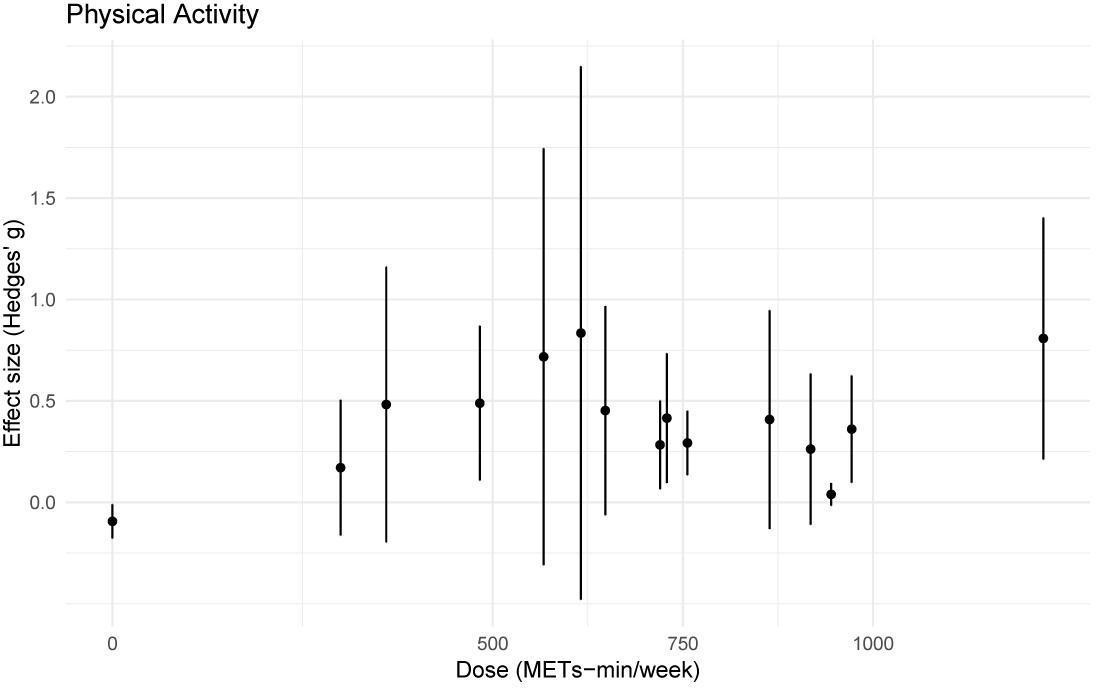


**Supplementary Figure 5.** “Split” NMA of overall exercise.

**Supplementary Table 2** shows the fit indices from each of the models fitted. For our data, restricted cubic splines show the best fit and were therefore used in subsequent analyses.

| **Model** | **DIC** | **SD** | **Deviance** | **Residual deviance** | **pD** |
| --- | --- | --- | --- | --- | --- |
| Emax (random | 65.5 | 0.148(0.0392, | 23.5 | 137.9 | 42.0 |
| treatment effects) |  | 0.258) |  |  |  |
| Restricted cubic splines (common treatment effects; 3 knots) | 54.4 | NA | 29.3 | 143.6 | 25.1 |
| Restricted cubic splines | 56.1 | 0.0957 (0.001, | 19.8 | 134.2 | 36.2 |
| (random treatment effects; 3 knots) |  | 0.217) |  |  |  |
| Restricted cubic spline | 57.8 | 0.0997 (0.00876, | 19.4 | 133.8 | 38.4 |

| (random treatment effects; 4 knots) |  | 0.222) |
| --- | --- | --- |
| Non-parametric monotonically up (random treatment effects) | 64.2 | NA NA NA 38.4 |

*Note.* DIC = Deviance Information Criterion; SD = Between-study Standard Deviation; pD: Number of estimated parameters; NA = Not Applicable. The SD is presented as the main value and (95% Credible Intervals).

Further to model fit indices, deviance plots showing the contribution of each data point to the residual deviance are also useful to confirm the robustness of model selection [[9]](https://paperpile.com/c/b8T1z2/q25h). Each data point should contribute about 1 to the posterior mean deviance, which indicates good model fit [[10]](https://paperpile.com/c/b8T1z2/6GsS). The deviance plot for overall (Supplementary File 6) and treatment effects (Supplementary File 7) confirm the robustness of our model selection (i.e., deviances < 2 except for few data points at 500 METs-min and 1000 METs-min in overall exercise and Mixed aerobic exercises, Walking/Jogging).


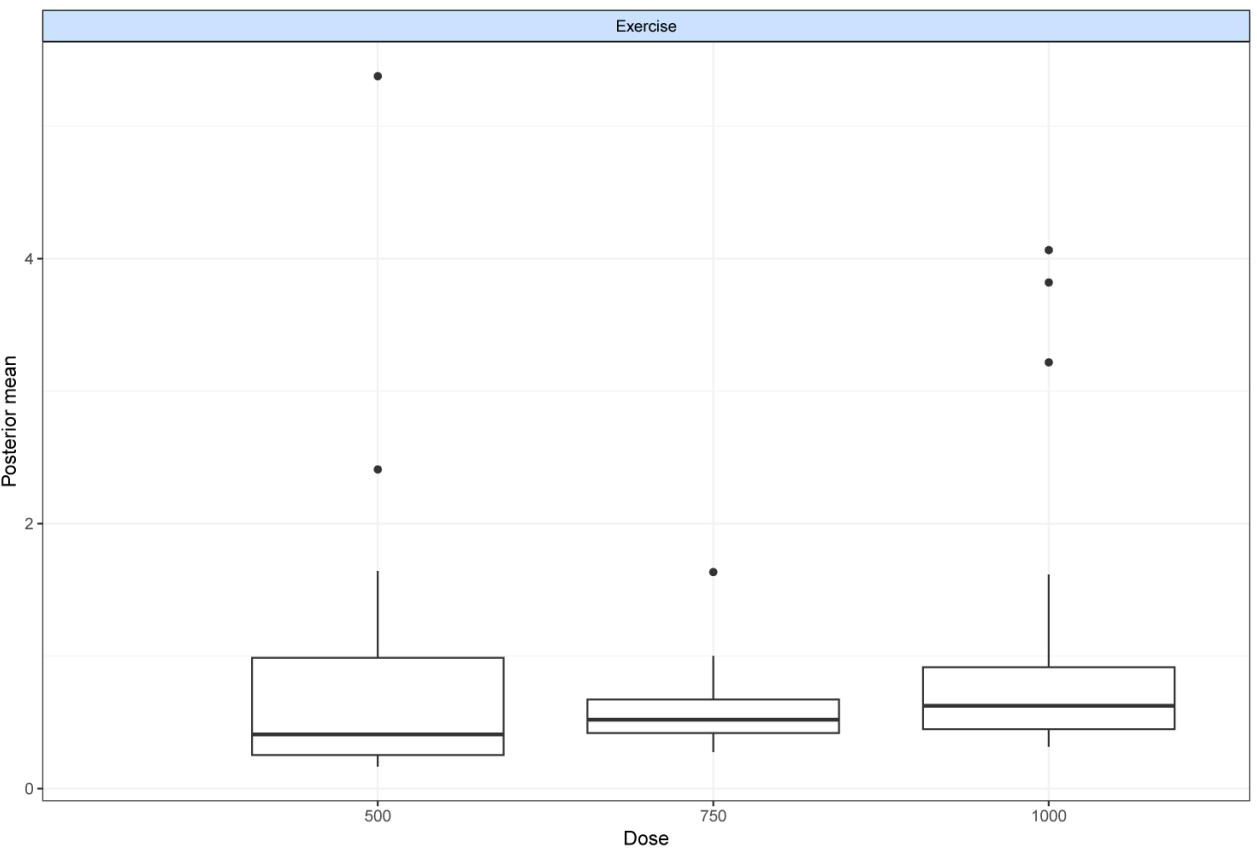


**Supplementary Figure 6**. Deviance plot at overall exercise level.


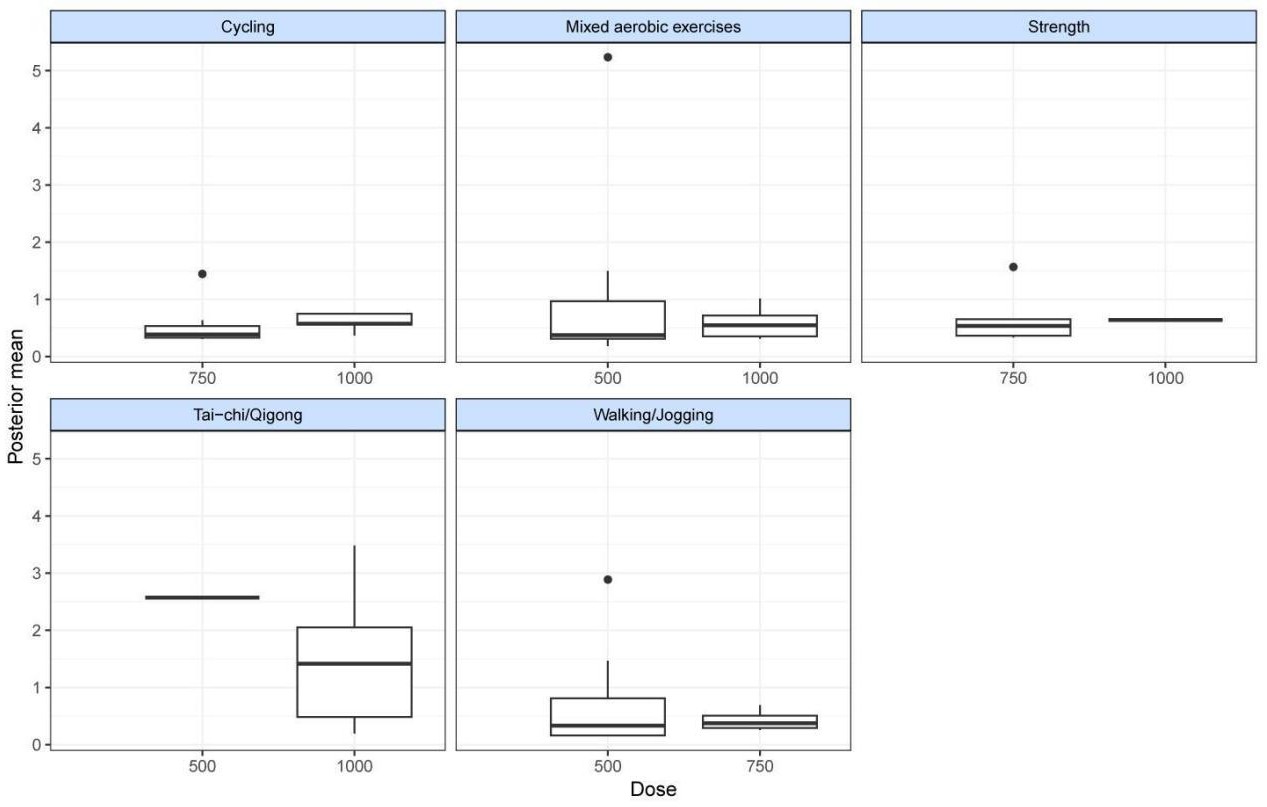


**Supplementary Figure 7.** Deviance plots at treatment-level.

## Supplementary File 4: League table, predicted responses

### Supplementary Table 3 is used to compare the relative effectiveness of different interventions, including usual care and various exercise interventions. It displays the estimated relative effects and 95% confidence intervals for each row intervention versus each column intervention, enabling analysis of differences in effectiveness among interventions.

|  | Usual care | 1_750 | 1_1000 | 2_500 | 2_1000 | 3_750 | 3_1000 | 4_500 | 4_1000 | 5_500 |
| --- | --- | --- | --- | --- | --- | --- | --- | --- | --- | --- |
| Usual care | 0 |  | | | | | | | | |
| 1_750 | **1.80**  (1.40,2.20) | 0 |  | | | | | | | |
| 1_1000 | **1.40**  (1.00,2.00) | **0.81**  (0.54,1.20) | 0 |  | | | | | | |
| 2_500 | **2.50**  (1.70,3.50) | **1.40**  (0.91,2.10) | **1.70**  (1.10,2.80) | 0 |  | | | | | |
| 2_1000 | **1.40**  (1.20,1.70) | **0.80**  (0.61,1.10) | **1.00**  (0.69,1.40) | **0.58**  (0.38,0.89) | 0 |  | | | | |
| 3_750 | **1.30**  (1.10,1.60) | **0.76**  (0.56,1.00) | **0.94**  (0.64,1.40) | **0.54**  (0.36,0.81) | **0.95**  (0.77,1.20) | 0 |  | | | |
| 3_1000 | **1.10**  (0.62,2.00) | **0.64**  (0.34,1.20) | **0.79**  (0.43,1.40) | **0.46**  (0.23,0.91) | **0.79**  (0.42,1.50) | **0.84**  (0.44,1.60) | 0 |  | | |
| 4_500 | **1.10**  (0.54,2.50) | **0.61**  (0.30,1.30) | **0.76**  (0.36,1.60) | **0.44**  (0.19,1.20) | **0.76**  (0.38,1.70) | **0.81**  (0.39,1.70) | **0.97**  (0.39,2.40) | 0 |  | |
| 4_1000 | **2.90**  (2.10,4.30) | **1.70**  (1.10,2.60) | **2.10**  (1.30,3.40) | **1.20**  (0.74,2.00) | **2.10**  (1.40,3.10) | **2.20**  (1.50,3.30) | **2.60**  (1.30,5.30) | **0.64**  (0.37,1.1) | 0 |  |
| 5_500 | **1.90**  (1.30,2.80) | **1.10**  (0.67,1.70) | **1.30**  (0.81,2.20) | **0.76**  (0.47,1.30) | **1.30**  (0.89,2.10) | **1.40**  (0.92,2.20) | **1.70**  (0.78,3.40) | **0.41**  (0.27,0.59) | **0.66**  (0.42,0.95) | 0 |

Notes. 1: Cycling; 2: Mixed aerobic exercises; 3: Strength; 4: Taichi/Qigong; 5: Walking/Jogging. The numbers that follow the intervention letter represent the dose

(METs-min/day). In this way and as example, the 1_500 intervention indicates the intervention of Cycling at 500 METs-min/week. To ease the interpretation of the table, the relative effects should be read as the row intervention vs. the column one.

### Supplementary Table 4. Predicted responses at 600,1200 METs per week.

Supplementary Table 4 shows the predicted effects for the lower and upper bound of WHO recommended level of physical activity [[11]](https://paperpile.com/c/b8T1z2/1osVX) as well as corresponding effects for the minimum WHO recommended level of physical activity [[11]](https://paperpile.com/c/b8T1z2/1osVX) for each type of intervention considered in our analysis.

**Agent**

| **Exercise Dose (METs-min/week)** | **SMD SD 95% CrI** | | |
| --- | --- | --- | --- |
| 600 | **0.55** | 3.92 | (0.23, 0.88) |
| 1200 | **0.58** | 2.78 | (0.34, 0.80) |
| 600 | **0.79** | 3.59 | (0.45, 1.12) |
| 1200 | **0.78** | 2.94 | (0.49, 1.04) |
| 600 | **0.35** | 4.57 | (-0.06, 0.76) |
| 1200 | **0.31** | 2.17 | (0.11, 0.50) |
| 600 | **0.01** | 4.32 | (-0.59, 0.62) |
| 1200 | **0.43** | 3.54 | (-0.04, 0.95) |
| 600 | **0.60** | 6.67 | (0.24, 1.06) |
| 1200 | **0.54** | 5.21 | (0.25, 0.89) |

**Cycling**

**Mixed aerobic exercises**

**Strength**

**Tai-chi/Qigong**

**Walking/Jogging**

**Supplementary *file 5: Characteristics of included studies***

### Supplementary Table 5. Overview of selected studies(k=15)

| **Study** | **Location** | **Age** | **Gender** | **Sample size** | **Depressive** | **Diagnostic** | **BMI, mean (S** | **Exercise intervention** |
| --- | --- | --- | --- | --- | --- | --- | --- | --- |
|  |  | **(mean or** | **(M/F)** | **(E/C)** | **Severity** | **criteria** | **D)(E/C)** |  |
|  |  | **range)** |  |  |  |  |  |  |
| **Buschert,2018** | **Germany** | **E：47.27** | **19/11** | **15/15** | **unspecified** | **ICD-10/BDI-** | **unspecified** | **Frequency:2-3 times/week** |
|  |  | **C：47.47** |  |  |  | **II; HAMD** |  | **Intensity:85%HRmax** |
|  |  |  |  |  |  |  |  | **Time:30mins** |
|  |  |  |  |  |  |  |  | **Session time:3-4weeks** |
| **Brush,2020** | **USA** | **E：20.26** | **49/17** | **35/31** | **mild to** | **MINI/BDI-II** | **23.87(4.23)/2** | **Frequency:3 times/week** |
|  |  | **C：20.19** |  |  | **moderate** |  | **3.64(4.58)** | **Intensity:40-65%HRR** |
|  |  |  |  |  |  |  |  | **Time:45 mins** |
|  |  |  |  |  |  |  |  | **Session time:8 weeks** |
| **Foley,2008** | **New** | **18-55** | **unspecified** | **10/13** | **unspecified** | **DSM-IV/BDI** | **unspecified** | **Frequency:3 times/week** |
|  | **Zealand** |  |  |  |  | **-II; MADRA** |  | **Intensity: moderate** |
|  |  |  |  |  |  | **S** |  | **Time:30-40 mins** |
|  |  |  |  |  |  |  |  | **Session time:12 weeks** |
| **Hoffman,2008** | **USA** | **E：51.9** | **153/49** | **104/49/49** | **unspecified** | **DSM-IV/HA** | **unspecified** | **Frequency:3 times/week** |
|  |  | **C¹：51.8** |  |  |  | **MD; BDI-II** |  | **Intensity:70-85%HRR** |
|  |  | **C²：51.2** |  |  |  |  |  | **Time:45 mins** |
|  |  |  |  |  |  |  |  | **Session time:16weeks** |
| **Imboden,2020** | **Switzerland** | **E：41.3** | **20/22** | **22/20** | **moderate to** | **ICD-10/HDR** | **25.9(5.4)/23.9** | **Frequency:3 times/week** |
|  |  | **C：38.3** |  |  | **severe** | **S; BDI** | **(4.8)** | **Intensity:60-75%HRmax** |
|  |  |  |  |  |  |  |  | **Time:45 mins** |
|  |  |  |  |  |  |  |  | **Session time:6weeks** |

| **Khatri,2001** | **USA** | **E/C:56.73** | **64/20** | **42/42** | **mild to** | **DSM-IV/HA** | **unspecified** | **Frequency:3 times/week** |
| --- | --- | --- | --- | --- | --- | --- | --- | --- |
|  |  |  |  |  | **moderate** | **MD; BDI-II** |  | **Intensity:70-85%HRR** |
|  |  |  |  |  |  |  |  | **Time:45 mins** |
|  |  |  |  |  |  |  |  | **Session time:16weeks** |
| **Krogh,2009** | **Denmark** | **E¹：41.9** | **122/43** | **55/55/55** | **mild to** | **ICD-10/HRS** | **unspecified** | **Frequency:2 times/week** |
|  |  | **E²：38.1** |  |  | **moderate** | **D; BDI** |  | **Intensity:50-70%RM** |
|  |  | **C：36.7** |  |  |  |  |  | **70-90%HRmax** |
|  |  |  |  |  |  |  |  | **Time:90 mins** |
|  |  |  |  |  |  |  |  | **Session time:16weeks** |
| **Krogh,2012** | **Denmark** | **E：39.7** | **77/38** | **56/59** | **mild to** | **MINI/HAM** | **26.2(6.2)/26.5** | **Frequency:3 times/week** |
|  |  | **C：43.4** |  |  | **moderate** | **D; BDI** | **(5.7)** | **Intensity:65-80%HRmax** |
|  |  |  |  |  |  |  |  | **Time:45 mins** |
|  |  |  |  |  |  |  |  | **Session time:12weeks** |
| **Krogh,2014** | **Denmark** | **41.3** | **26/53** | **41/38** | **mild to** | **MINI/HAM** | **25.8(6.4)/25.2** | **Frequency:3 times/week** |
|  |  |  |  |  | **moderate** | **D** | **(5.1)** | **Intensity:80%HRmax** |
|  |  |  |  |  |  |  |  | **Time:45 mins** |
|  |  |  |  |  |  |  |  | **Session time:12weeks** |
| **Lavretsky,2011** | **USA** | **E: 69.1** | **45/28** | **36/37** | **moderate to** | **HDRS** | **unspecified** | **Frequency:1 times/week** |
|  |  | **C: 72.0** |  |  | **severe** |  |  | **Intensity: mild** |
|  |  |  |  |  |  |  |  | **Time:120mins** |
|  |  |  |  |  |  |  |  | **Session time:10weeks** |
| **Neviani,2017** | **Italy** | **75.2** | **35/86** | **42/37/42** | **mild to** | **DSM-IV/HA** | **25.8(3.3)/25.2** | **Frequency:3 times/week** |
|  |  |  |  |  | **moderate** | **MD** | **(3.7)/26.7(3.8** | **Intensity:60-85%HRmax** |
|  |  |  |  |  |  |  | **)** | **Time:60mins** |
|  |  |  |  |  |  |  |  | **Session time:24weeks** |
| **Oertel,2014** | **Germany** | **E：36.63** | **11/11** | **8/6/8** | **unspecified** | **DSM-IV/BDI** | **unspecified** | **Frequency:3 times/week** |

|  |  | **C¹：20.19** |  |  |  | **-II** |  | **Intensity:60-70%HRmax** |
| --- | --- | --- | --- | --- | --- | --- | --- | --- |
|  |  | **C²：42.21** |  |  |  |  |  | **Time:45 mins** |
|  |  |  |  |  |  |  |  | **Session time:4weeks** |
| **Olson,2017** | **USA** | **E：21.0** | **24/6** | **15/15** | **unspecified** | **DSM-IV/BDI** | **unspecified** | **Frequency:3 times/week** |
|  |  | **C：21.2** |  |  |  | **-II** |  | **Intensity:40-65%HRR** |
|  |  |  |  |  |  |  |  | **Time:45 mins** |
|  |  |  |  |  |  |  |  | **Session time:8weeks** |
| **Sharma,2006** | **India** | **E: 31.87** | **11/19** | **15/15** | **unspecified** | **DSM-IV/HA** | **unspecified** | **Frequency:3 times/week** |
|  |  | **C: 31.67** |  |  |  | **M-D** |  | **Intensity: mild** |
|  |  |  |  |  |  |  |  | **Time:30 mins** |
|  |  |  |  |  |  |  |  | **Session time:8weeks** |
| **Zhang,2022** | **China** | **E：47.20** | **4/35** | **20/19** | **unspecified** | **BDI-II** | **22.83(3.60)/** | **Frequency:2 times/week** |
|  |  | **C：54.16** |  |  |  |  | **23.59(3.42)** | **Intensity:85%HRmax** |
|  |  |  |  |  |  |  |  | **Time:90mins** |
|  |  |  |  |  |  |  |  | **Session time:12weeks** |

Note. k = number of included studies; %=percentage; E=experimental group; C=control group; C¹=first control group; C²=second control group;

SD=standard deviation; M=male; F=female; MINI = The Mini-International neuropsychiatric interview; DSM‑ IV = The Diagnostic and Statistical Manual of Mental Disorders, Fourth Edition; ICD-10 = International Classification of Diseases, Tenth Revision; BDI = Beck Depression Inventory; BDI-II = Beck Depression Inventory-II; MADRAS = the Montgomery-Åsberg Depression Rating Scale; HAMD/HDRS/HRSD = Hamilton Depression Rating Scale; Diagnostic criteria/instruments = Diagnostic criteria and depression scales.

**Supplementary *file 6: Quality assessment of included studies***

### Supplementary Table 6 presents the quality assessment results of the included studies. Using a quality assessment sheet, it lists each study's compliance with specific criteria (e.g., randomization, allocation concealment, baseline similarity), marked as "Y" (Yes) or "N" (No). The "TS" column indicates the total score for each study, and "OSQ" reflects the overall study quality (High or Moderate). The final row shows the mean score, providing a quantitative evaluation of the overall quality.

| **Reference** | **Year** | **1** | **2** | **3** | **4** | **5** | **6** | **7** | **8** | **9** | **10** | **11** | **TS** | **OSQ** |
| --- | --- | --- | --- | --- | --- | --- | --- | --- | --- | --- | --- | --- | --- | --- |
| **Brush et al.** | **2020** | **Y** | **Y** | **N** | **Y** | **N** | **N** | **Y** | **N** | **Y** | **Y** | **Y** | **6** | **Moderate** |
| **Buschert et al.** | **2018** | **Y** | **Y** | **N** | **Y** | **N** | **N** | **N** | **N** | **Y** | **Y** | **Y** | **5** | **Moderate** |
| **Foley et al.** | **2008** | **Y** | **Y** | **N** | **Y** | **N** | **N** | **N** | **N** | **N** | **Y** | **Y** | **4** | **Poor** |
| **Hoffman et al.** | **2008** | **Y** | **Y** | **Y** | **Y** | **N** | **N** | **Y** | **N** | **Y** | **Y** | **Y** | **7** | **Moderate** |
| **Imboden et al.** | **2020** | **Y** | **Y** | **N** | **Y** | **Y** | **N** | **Y** | **N** | **Y** | **Y** | **Y** | **7** | **Moderate** |
| **Khatri et al.** | **2001** | **Y** | **Y** | **N** | **Y** | **N** | **N** | **Y** | **N** | **Y** | **Y** | **Y** | **6** | **Moderate** |
| **Krogh et al.** | **2009** | **Y** | **Y** | **Y** | **Y** | **N** | **N** | **Y** | **N** | **Y** | **Y** | **Y** | **7** | **Moderate** |
| **Krogh et al.** | **2012** | **Y** | **Y** | **Y** | **Y** | **N** | **N** | **Y** | **Y** | **Y** | **Y** | **Y** | **8** | **High** |
| **Krogh et al.** | **2014** | **Y** | **Y** | **Y** | **N** | **N** | **N** | **Y** | **N** | **N** | **Y** | **Y** | **5** | **Moderate** |
| **Lavretsky et al.** | **2011** | **Y** | **Y** | **Y** | **Y** | **N** | **N** | **Y** | **Y** | **Y** | **Y** | **Y** | **8** | **High** |
| **Neviani et al.** | **2017** | **Y** | **Y** | **Y** | **Y** | **N** | **N** | **Y** | **Y** | **Y** | **Y** | **Y** | **8** | **High** |
| **Oertel-Knochel et al.** | **2014** | **Y** | **Y** | **N** | **Y** | **N** | **N** | **Y** | **N** | **Y** | **Y** | **Y** | **6** | **Moderate** |
| **Olson et al.** | **2017** | **Y** | **Y** | **N** | **Y** | **N** | **N** | **N** | **N** | **N** | **Y** | **Y** | **4** | **Poor** |
| **Sharma et al** | **2006** | **Y** | **Y** | **N** | **Y** | **N** | **N** | **N** | **Y** | **Y** | **Y** | **Y** | **7** | **Moderate** |
| **Zhang et al.** | **2022** | **Y** | **Y** | **N** | **Y** | **N** | **N** | **N** | **Y** | **Y** | **Y** | **Y** | **6** | **Moderate** |
| **Mean score** |  |  |  |  |  |  |  |  |  |  |  |  | **6.25** |  |

1 Eligibility criteria, 2 allocation of randomization, 3 concealed allocation, 4 similarity baseline, 5 subject blinding, 6 therapist blinding, 7 assessor blinding, 8 more than 85% retention, 9 intention-to-treat analysis, 10 between-group comparisons, 11 point and variability measures, TS total score, OSQ overall study quality, Y explicitly described and present in details, N absent, inadequately described, or unclear

**Supplementary *file 7: Moderation analysis***

The following three charts shows the dose-response relationship between exercise and change in cognition after exploring the relationship between certain influencing factors (i.e., sex, age and depressive severity) and cognitive function.


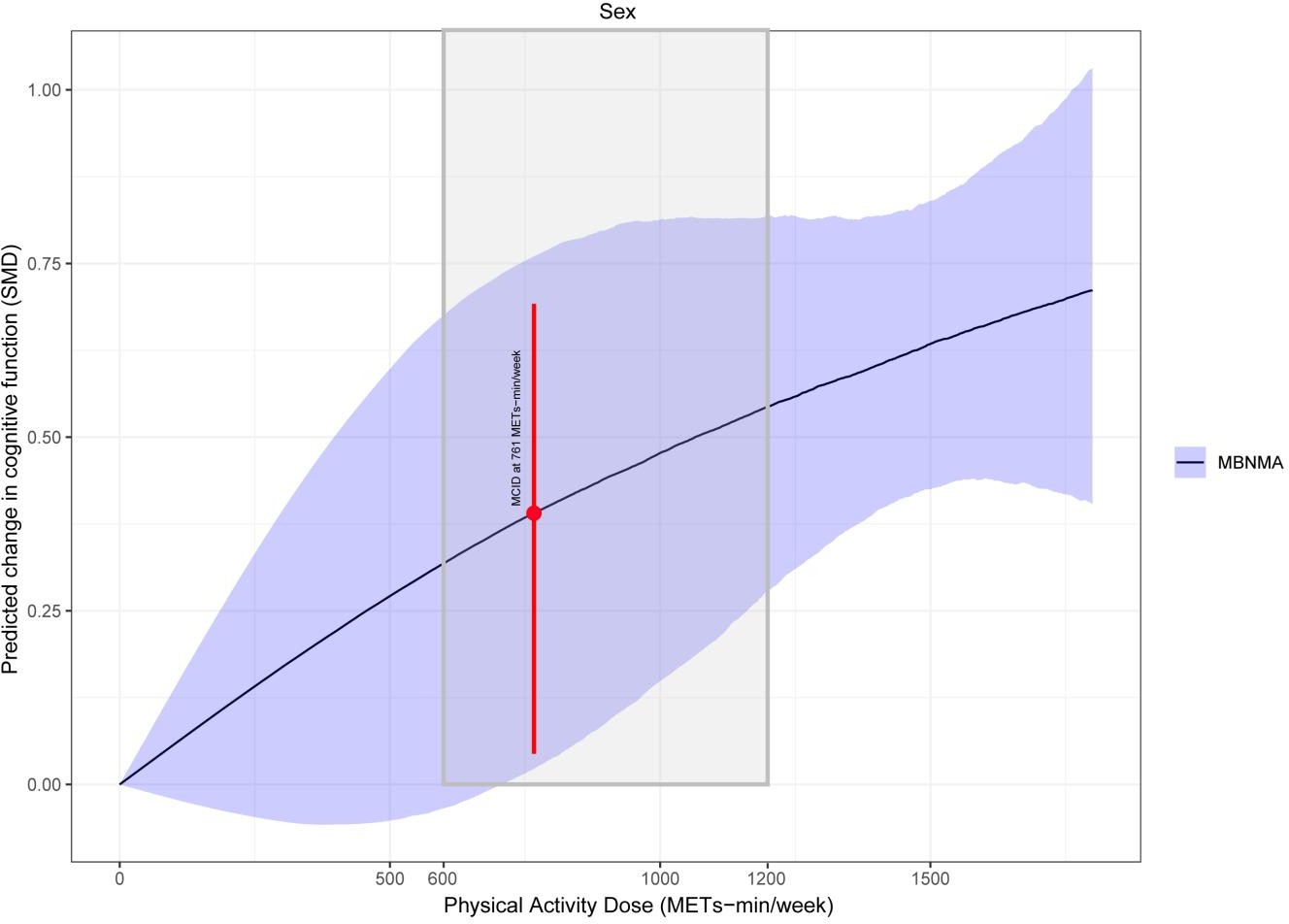


**Supplementary Figure 8.** Dose-response curve between exercise and changes in cognitive function (proportion of females: ≥ 50%).


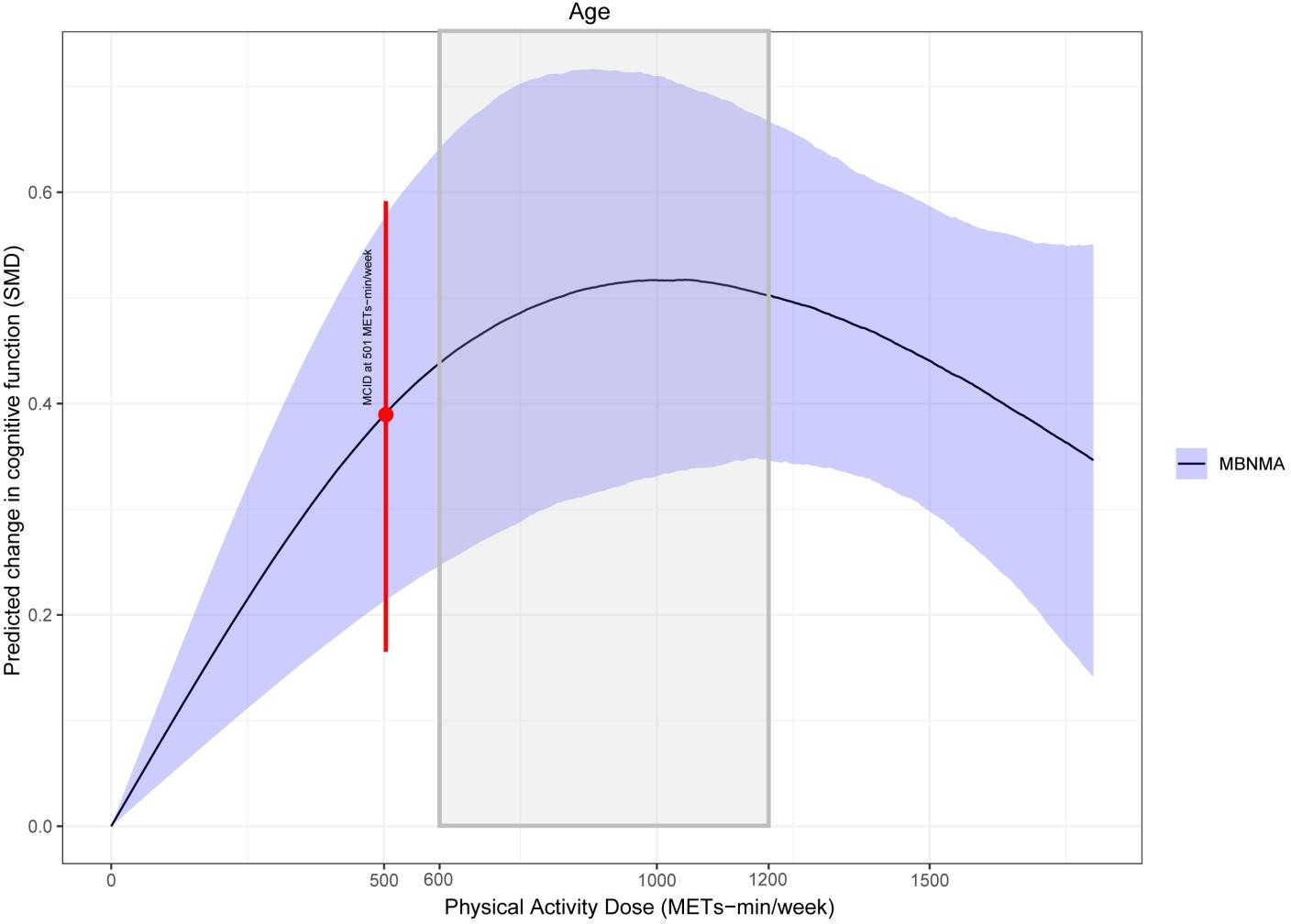


**Supplementary Figure 9.** Dose-response curve between exercise and changes in cognitive function (age: 18-44 year olds).

**Supplementary *file 8: Sensitivity analysis***

Supplementary Figure 11 shows the dose-response relationship between exercise and change in cognition after excluding studies with an unclear and high risk of bias.


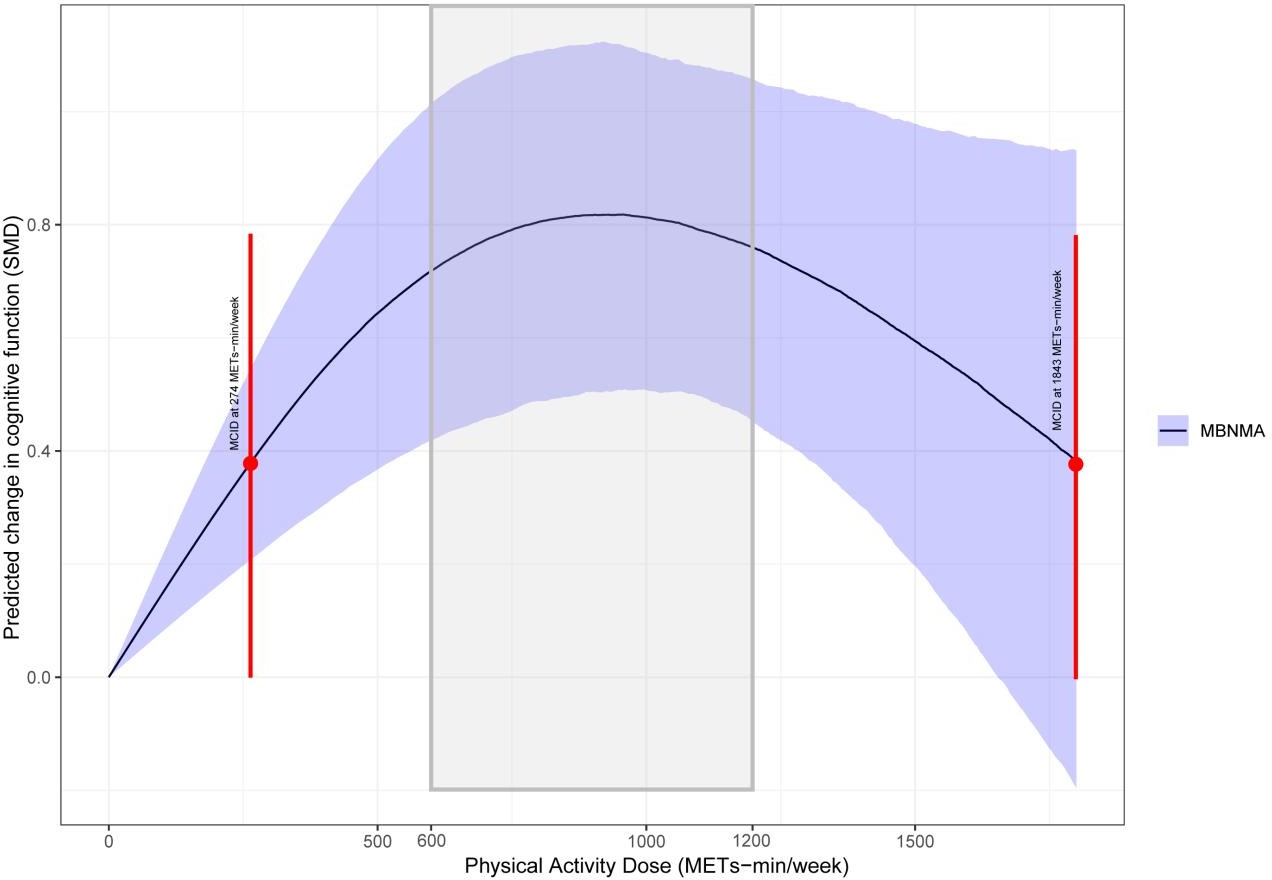


**Supplementary Figure 10.** Dose-response curve between exercise and changes in cognitive function only including studies with low risk of bias.

# Supplementary *file 9:* Risk of bias

#
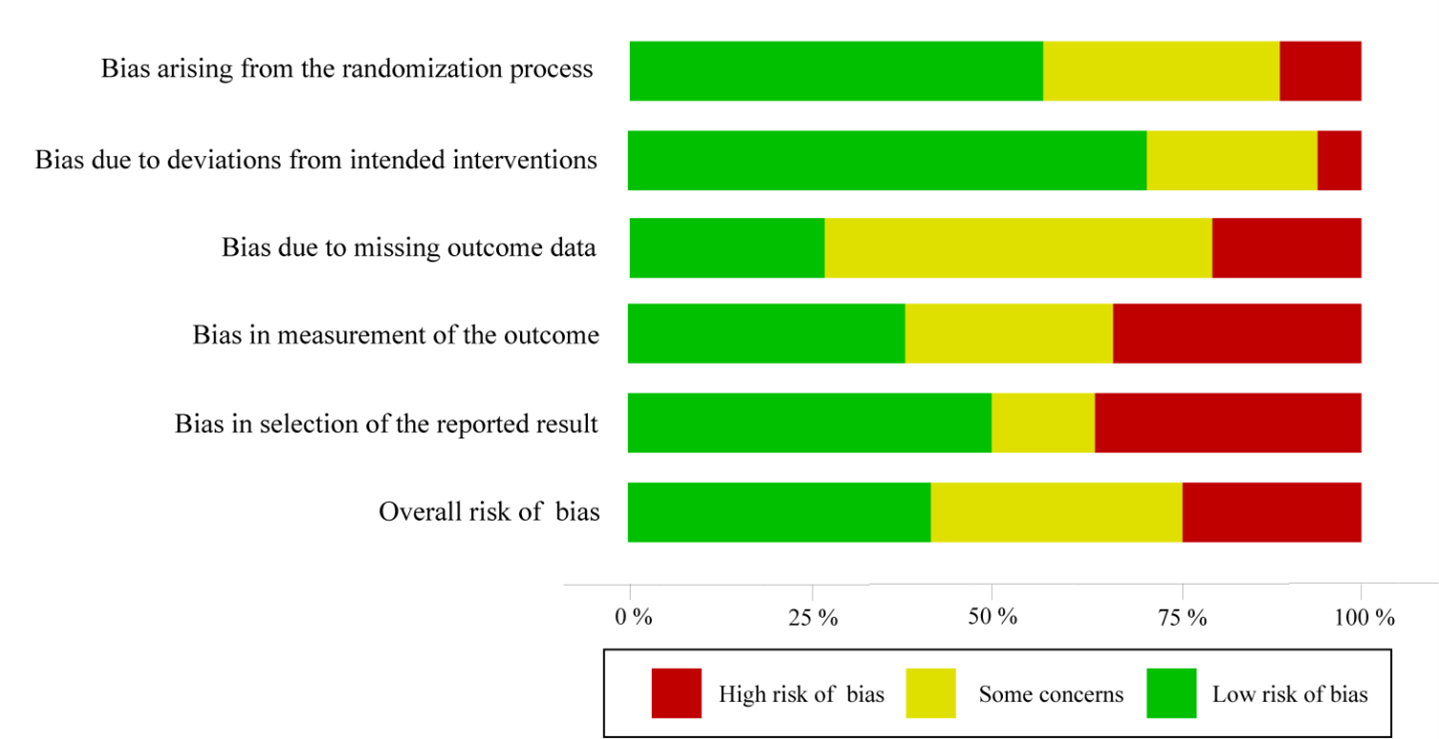
Supplementary Figure 11 is used to assess the risk of bias in various domains of the study using the Cochrane Risk of Bias Tool. It illustrates the distribution of bias across domains, including bias arising from the randomization process, deviations from intended interventions, missing outcome data, outcome measurement, and selection of the reported result. The color coding (green for low risk of bias, yellow for some concerns, and red for high risk of bias) provides a clear visual representation of the severity and distribution of bias.

# Supplementary Figure 11. Cochrane Risk of Bias Tool.

# 24

# References

1. [Donegan S, Williamson P, D’Alessandro U,](http://paperpile.com/b/b8T1z2/kvl9) [*et al.*](http://paperpile.com/b/b8T1z2/kvl9) [Assessing key assumptions of network](http://paperpile.com/b/b8T1z2/kvl9) [meta-analysis: a review of methods.](http://paperpile.com/b/b8T1z2/kvl9) [*Res Synth Methods*](http://paperpile.com/b/b8T1z2/kvl9) [2013;4:291–323. doi:](http://paperpile.com/b/b8T1z2/kvl9)[10.1002/jrsm.1085](http://dx.doi.org/10.1002/jrsm.1085)
2. [Watt J, Tricco AC, Straus S,](http://paperpile.com/b/b8T1z2/gaquA) [*et al.*](http://paperpile.com/b/b8T1z2/gaquA) [Research Techniques Made Simple: Network Meta-Analysis.](http://paperpile.com/b/b8T1z2/gaquA) [*J*](http://paperpile.com/b/b8T1z2/gaquA) [*Invest Dermatol*](http://paperpile.com/b/b8T1z2/gaquA) [2019;139:4–12.e1. doi:](http://paperpile.com/b/b8T1z2/gaquA)[10.1016/j.jid.2018.10.028](http://dx.doi.org/10.1016/j.jid.2018.10.028)
3. [ter Veer E, van Oijen MGH, van Laarhoven HWM. The Use of (Network) Meta-Analysis in](http://paperpile.com/b/b8T1z2/YP82p) [Clinical Oncology.](http://paperpile.com/b/b8T1z2/YP82p) [*Front Oncol*](http://paperpile.com/b/b8T1z2/YP82p) [2019;9:822. doi:](http://paperpile.com/b/b8T1z2/YP82p)[10.3389/fonc.2019.00822](http://dx.doi.org/10.3389/fonc.2019.00822)
4. [Wheeler DC, Hickson DA, Waller LA. Assessing local model adequacy in Bayesian hierarchical](http://paperpile.com/b/b8T1z2/jIaJT) [models using the partitioned deviance information criterion. Computational Statistics & Data](http://paperpile.com/b/b8T1z2/jIaJT) [Analysis. 2010;54:1657–71. doi:](http://paperpile.com/b/b8T1z2/jIaJT)[10.1016/j.csda.2010.01.025](http://dx.doi.org/10.1016/j.csda.2010.01.025)
5. [White IR, Barrett JK, Jackson D,](http://paperpile.com/b/b8T1z2/VBvcg) [*et al.*](http://paperpile.com/b/b8T1z2/VBvcg) [Consistency and inconsistency in network meta-analysis:](http://paperpile.com/b/b8T1z2/VBvcg) [model estimation using multivariate meta-regression.](http://paperpile.com/b/b8T1z2/VBvcg) [*Res Synth Methods*](http://paperpile.com/b/b8T1z2/VBvcg) [2012;3:111–25.](http://paperpile.com/b/b8T1z2/VBvcg) [doi:](http://paperpile.com/b/b8T1z2/VBvcg)[10.1002/jrsm.1045](http://dx.doi.org/10.1002/jrsm.1045)
6. [Cipriani A, Higgins JPT, Geddes JR,](http://paperpile.com/b/b8T1z2/hbtK1) [*et al.*](http://paperpile.com/b/b8T1z2/hbtK1) [Conceptual and technical challenges in network](http://paperpile.com/b/b8T1z2/hbtK1) [meta-analysis.](http://paperpile.com/b/b8T1z2/hbtK1) [*Ann Intern Med*](http://paperpile.com/b/b8T1z2/hbtK1) [2013;159:130–7. doi:](http://paperpile.com/b/b8T1z2/hbtK1)[10.7326/0003-4819-159-2-201307160-00008](http://dx.doi.org/10.7326/0003-4819-159-2-201307160-00008)
7. [Salanti G, Del Giovane C, Chaimani A,](http://paperpile.com/b/b8T1z2/El6R) [*et al.*](http://paperpile.com/b/b8T1z2/El6R) [Evaluating the quality of evidence from a network](http://paperpile.com/b/b8T1z2/El6R) [meta-analysis.](http://paperpile.com/b/b8T1z2/El6R) [*PLoS One*](http://paperpile.com/b/b8T1z2/El6R) [2014;9:e99682. doi:](http://paperpile.com/b/b8T1z2/El6R)[10.1371/journal.pone.0099682](http://dx.doi.org/10.1371/journal.pone.0099682)
8. [van Valkenhoef G, Dias S, Ades AE,](http://paperpile.com/b/b8T1z2/RQrTH) [*et al.*](http://paperpile.com/b/b8T1z2/RQrTH) [Automated generation of node-splitting models for](http://paperpile.com/b/b8T1z2/RQrTH) [assessment of inconsistency in network meta-analysis.](http://paperpile.com/b/b8T1z2/RQrTH) [*Res Synth Methods*](http://paperpile.com/b/b8T1z2/RQrTH) [2016;7:80–93.](http://paperpile.com/b/b8T1z2/RQrTH) [doi:](http://paperpile.com/b/b8T1z2/RQrTH)[10.1002/jrsm.1167](http://dx.doi.org/10.1002/jrsm.1167)
9. [Pedder H. MBNMAdose: An R package for incorporating dose-response information into](http://paperpile.com/b/b8T1z2/q25h) [Network Meta-Analysis. In:](http://paperpile.com/b/b8T1z2/q25h) [*Evidence Synthesis and Meta-Analysis in R Conference 2021*. 2021.](http://paperpile.com/b/b8T1z2/q25h) [https://research-information.bris.ac.uk/en/publications/mbnmadose-an-r-package-for-incorporatin](https://research-information.bris.ac.uk/en/publications/mbnmadose-an-r-package-for-incorporating-dose-response-informatio) [g-dose-response-informatio](https://research-information.bris.ac.uk/en/publications/mbnmadose-an-r-package-for-incorporating-dose-response-informatio)
10. [Dias S, Sutton AJ, Ades AE,](http://paperpile.com/b/b8T1z2/6GsS) [*et al.*](http://paperpile.com/b/b8T1z2/6GsS) [Evidence synthesis for decision making 2: a generalized linear](http://paperpile.com/b/b8T1z2/6GsS) [modeling framework for pairwise and network meta-analysis of randomized controlled trials.](http://paperpile.com/b/b8T1z2/6GsS) [*Med*](http://paperpile.com/b/b8T1z2/6GsS) [*Decis Making*](http://paperpile.com/b/b8T1z2/6GsS) [2013;33:607–17. doi:](http://paperpile.com/b/b8T1z2/6GsS)[10.1177/0272989X12458724](http://dx.doi.org/10.1177/0272989X12458724)
11. [Bull FC, Al-Ansari SS, Biddle S,](http://paperpile.com/b/b8T1z2/1osVX) [*et al.*](http://paperpile.com/b/b8T1z2/1osVX) [World Health Organization 2020 guidelines on ph](http://paperpile.com/b/b8T1z2/1osVX) [ysical activity and sedentary behaviour.](http://paperpile.com/b/b8T1z2/1osVX) [*Br J Sports Med*](http://paperpile.com/b/b8T1z2/1osVX) [2020;54:1451–62. doi:](http://paperpile.com/b/b8T1z2/1osVX)[10.1136/bjs](http://dx.doi.org/10.1136/bjsports-2020-102955) [ports-2020-102955](http://dx.doi.org/10.1136/bjsports-2020-102955)

25
